# TSHR‐Targeting Nucleic Acid Aptamer Treats Graves' Ophthalmopathy via Novel Allosteric Inhibition

**DOI:** 10.1002/advs.202505586

**Published:** 2025-10-07

**Authors:** Yanchen Zhang, Ende Wu, Weibin Liu, Ling Zeng, Neng Ling, Hongmei Wang, Zhixing Li, Shuang Yao, Tonghe Pan, Xuanwen Li, Yate Huang, Xiaojing Li, Yunhai Tu, Wentao Yan, Jianzhang Wu, Mao Ye, Wencan Wu

**Affiliations:** ^1^ State Key Laboratory of Eye Health Eye Hospital Wenzhou Medical University Wenzhou Zhejiang 325027 China; ^2^ Molecular Science and Biomedicine Laboratory (MBL) State Key Laboratory of Chemo and Biosensing Hunan Research Center of the Basic Discipline for Cell Signaling College of Biology College of Chemistry and Chemical Engineering Aptamer Engineering Center of Hunan Province Hunan University Changsha Hunan 410082 China; ^3^ Oujiang Laboratory (Zhejiang Lab for Regenerative Medicine Vision and Brain Health) Wenzhou Zhejiang 325000 China; ^4^ Zhejiang Key Laboratory of Key Technologies for Visual Pathway Reconstruction Eye Hospital Wenzhou Medical University Wenzhou Zhejiang 325027 China

**Keywords:** allosteric inhibition, Graves' ophthalmopathy, nucleic acid aptamer, targeted therapy, thyrotropin receptor

## Abstract

Graves' ophthalmopathy (GO) is an autoimmune disorder marked by orbital inflammation and tissue remodeling, leading to irreversible disfigurement and vision loss. The current first‐line glucocorticoid therapy remains palliative, underscoring the critical need for mechanism‐based interventions. Autoantibodies against thyrotropin receptor (TSHR) in GO patients highlight its therapeutic potential, yet TSHR inhibitor development faces challenges, including low potency, off‐target effects, and mechanistic constraints. To overcome this therapeutic void, YC3, a TSHR‐targeting nucleic acid aptamer, has been developed through an innovative approach that combines protein‐targeting cell‐SELEX with functional selection. YC3 exhibits nanomolar affinity alongside robust pharmacodynamic efficacy. In vitro, YC3 significantly reverses thyroid‐stimulating antibodies (TSAbs)‐driven hyperactivation in primary human orbital fibroblasts, thereby suppressing pathogenic hallmarks of fibroblasts. In vivo, therapeutic administration of YC3 significantly alleviates ocular symptoms in a GO mouse model. Mechanistic investigations reveal that YC3 binds to a previously unidentified allosteric site within the leucine‐rich repeat domain of TSHR, consequently inhibiting receptor activation. Collectively, this study not only identifies YC3 as a promising TSHR‐targeting therapeutic candidate but also unveils a novel allosteric site for next‐generation inhibitors. These findings highlight the potential of aptamers in both dissecting receptor mechanisms and uncovering cryptic druggable sites, thereby bridging structural biology with targeted drug development.

## Graphical abstract

This study presents YC3, a novel inhibitory TSHR‐targeting aptamer, as a promising therapeutic for Graves' ophthalmopathy (GO). YC3 suppresses pathological phenotypes in human orbital fibroblasts and improves outcomes in GO mice by binding to a previously unidentified allosteric site on TSHR, demonstrating the potential of aptamers in advancing targeted therapy, discovering cryptic druggable sites, and uncovering receptor mechanisms.

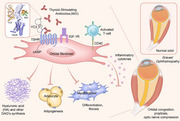

## 1 Introduction

Graves’ ophthalmopathy (GO), also known as thyroid eye disease (TED), is an orbit‐specific autoimmune disorder. It is characterized by proptosis, eyelid retraction, and restrictive strabismus, which can result in disfigurement, vision impairment, and a diminished quality of life.^[^ ^1^, ^2^ ^]^ GO is most commonly associated with hyperthyroidism due to Graves' disease (GD) and has an incidence of 16 per 100,000 in females and 2.9 per 100,000 in males.^[^ ^3^, ^4^, ^5^ ^]^ Current management prioritizes drug therapy, with intravenous glucocorticoids serving as the first‐line treatment for moderate‐to‐severe GO due to their anti‐inflammatory effect.^[^ ^6^ ^]^ However, these therapies are largely palliative, failing to address the underlying autoimmune pathogenesis, and their broad immunosuppressive role carries risks of significant adverse effects.^[^ ^7^ ^]^ In addition, emerging biological agents, including the monoclonal antibodies Teprotumumab, Rituximab, and Tocilizumab, have emerged as the second‐line targeted therapies for refractory or glucocorticoid‐resistant GO.^[^ ^4^ ^]^ Despite their potential to modulate specific immune pathways, these therapies come with limitations. Variability in efficacy across different patient subgroups, along with high costs, concerns regarding immunogenicity, and safety issues, complicates the use of these therapies.^[^ ^8^ ^]^ Collectively, these challenges contribute to reduced patient acceptance and adherence, underscoring the need for safer, more cost‐effective, and durable mechanism‐driven therapeutic strategies.

The dysregulated thyrotropin receptor (TSHR) signaling pathway plays a central role in the pathogenesis and progression of GO. TSHR, a G protein‐coupled receptor (GPCR) predominantly expressed in thyroid follicular cells, governs thyroid hormone synthesis and secretion via activation by thyrotropin (TSH), thereby maintaining metabolic homeostasis.^[^ ^9^, ^10^ ^]^ Beyond its canonical thyroidal role, TSHR is also expressed in extrathyroidal tissues such as the brain, thymus, and orbit, where it contributes to immune modulation.^[^ ^11^, ^12^ ^]^ In GO, TSHR expression is markedly upregulated in orbital tissues, including fibroblasts, adipocytes, and extraocular muscles, and its hyperactivity is driven by thyroid‐stimulating antibodies (TSAbs).^[^ ^13^, ^14^, ^15^, ^16^ ^]^ These autoantibodies, commonly found in GO patients, show a strong correlation between their serum TSAb levels and the clinical activity and severity of GO.^[^ ^17^, ^18^, ^19^ ^]^ The TSAbs bind to the extracellular domain of TSHR, structurally mimicking TSH and inducing conformational activation of the receptor. Unlike the transient signaling induced by TSH, TSAbs cause sustained receptor hyperactivation, leading to dysregulated downstream pathways.^[^ ^12^, ^20^, ^21^, ^22^ ^]^ This prolonged activation results in hyperactivation of fibroblasts that secrete proinflammatory cytokines and hyaluronan, perpetuating tissue inflammation and edema. Furthermore, this aberrant signaling reprograms orbital fibroblasts into pathogenic effector cells, such as myofibroblasts or adipocytes, contributing to abnormal orbital tissue remodeling.^[^ ^23^, ^24^, ^25^, ^26^, ^27^, ^28^ ^]^ Moreover, similar pathological processes have been observed in the TSHR‐immunized GO mouse model, supporting the “Pandora's box” role of TSHR.^[^ ^29^, ^30^ ^]^

Dysregulation of the TSHR underpins multiple thyroid‐related pathologies, including GD, GO, hypothyroidism, and thyroid cancer, positioning TSHR as a high‐value therapeutic target.^[^ ^12^ ^]^ Over the past decades, various TSHR inhibitors such as small molecules, monoclonal antibodies, and peptides have emerged.^[^ ^12^, ^31^, ^32^ ^]^ However, most exhibit suboptimal characteristics like inadequate potency, off‐target effects, and poor pharmacokinetics. Only the binding mechanism of the orthosteric inhibitor K1‐70 monoclonal antibody has been elucidated, leaving the molecular interactions of other inhibitors undefined.^[^ ^10^ ^]^ This knowledge gap, combined with the clinical demand for TSHR inhibitors, underscores the urgent necessity to develop well‐characterized and effective TSHR inhibitors based on a clear understanding of mechanisms.

Aptamers are an innovative class of therapeutic molecules consisting of single‐stranded nucleic acids, typically 20 to 100 nucleotides in length. They form a unique 3D structure that enables high‐affinity and specific binding to their target.^[^ ^33^ ^]^ These molecules offer distinct advantages over conventional therapeutics, including ease of synthesis and modification, low toxicity, cost‐effectiveness, and lack of immunogenicity.^[^ ^34^, ^35^ ^]^ The clinical potential of aptamers is highlighted by FDA‐approved therapies like Pegaptanib and Zimura.^[^ ^36^ ^]^

In this study, we employed an innovative tandem approach combining protein‐targeted cell‐SELEX with specific functional selection to develop YC3, a TSHR‐targeting inhibitory aptamer. Our findings indicate that YC3 exerts its inhibitory effects by binding to a previously unidentified allosteric site on the TSHR. This novel mechanism, along with biological activity in both in vitro and in vivo systems, demonstrates YC3's therapeutic potential for GO.

## 2 Results

### 2.1 Screening and Identification of TSHR‐targeting Aptamers with Inhibitory Function

**Figure 1:**
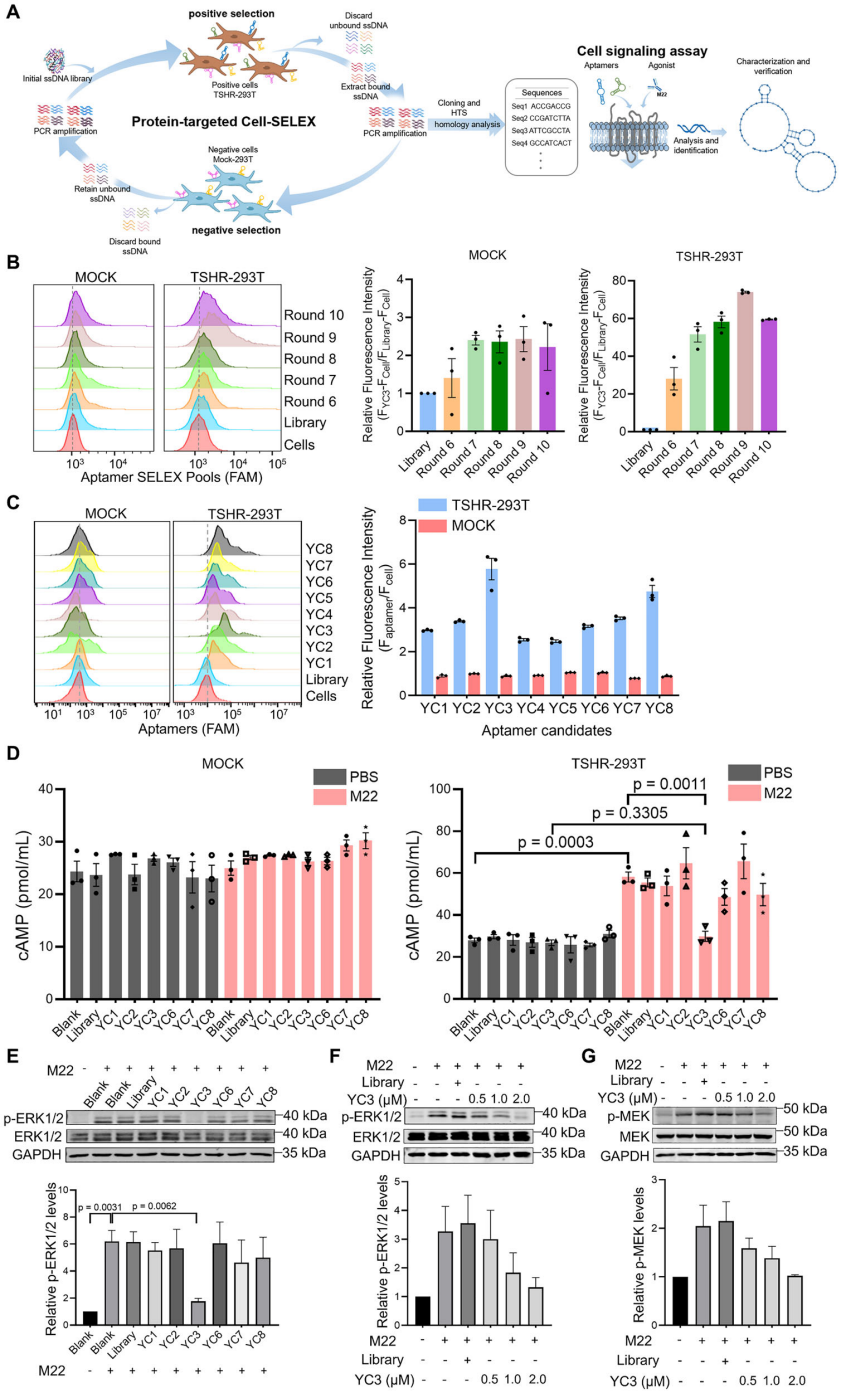
Screening of TSHR‐targeting DNA aptamers with inhibitory function. A) Schematic illustration of the aptamer inhibitors selection strategy for TSHR. B) Binding assays of the selected pools with MOCK and TSHR‐293T cells by flow cytometry. The FAM‐labeled initial Library was used as the control. Quantitative analysis of the relative fluorescence intensity of the aptamer candidates in TSHR‐293T and MOCK cells (n = 3). C) The binding of aptamer candidates (250 nM) to MOCK and TSHR‐293T cells was analyzed by flow cytometry. Quantitative analysis of the relative fluorescence intensity of the aptamer candidates in TSHR‐293T and MOCK cells (n = 3). D) The intracellular cAMP levels in MOCK and TSHR‐293T cells, treated with or without aptamer candidates and M22, were measured by ELISA (n = 3). E) Representative immunoblots/densitometric quantitative analysis of phospho‐ERK1/2 protein level and ERK1/2 protein level in TSHR‐293T cells stimulated by M22 and treated with aptamer candidates. GAPDH was used as a loading control (n = 3). F) Representative immunoblots/densitometric quantitative analysis of phospho‐ERK1/2 protein level and ERK1/2 protein level in TSHR‐293T cells stimulated by M22 and treated with a series of concentrations of YC3. GAPDH was used as a loading control (n = 3). G) Representative immunoblots/densitometric quantitative analysis of phospho‐MEK protein level and MEK protein level in TSHR‐293T cells stimulated by M22 and treated with a series of concentrations of YC3. GAPDH was used as a loading control (n = 3). All data are represented as the mean ± SEM. Two‐tailed unpaired Student's t‐tests were used to calculate P values (E). Accurate P values are listed in the figures.

To screen TSHR‐targeting aptamers with inhibitory function, we employed a tandem process combining protein‐targeted cell‐SELEX and a specific functional selection process (Figure 1A). For protein‐targeted cell‐SELEX, HEK293T cells stably expressing TSHR (TSHR‐293T) were used as positive cells, while HEK293T cells transfected with an empty vector (MOCK) served as negative controls (Figure S1A, Supporting Information). The expression levels of both TSHR mRNA and protein were high in TSHR‐293T cells, whereas no detectable expression was observed in MOCK cells, as determined by qPCR, flow cytometry, and western blotting (Figure S1B–D, Supporting Information). Subsequently, cell‐SELEX was conducted using TSHR‐293T cells for positive selection and MOCK cells for negative selection. The enrichment of the ssDNA sequences in different rounds was monitored by flow cytometry. With each subsequent screening round, the fluorescence intensity of the TSHR‐293T cells progressively increased, whereas that of the MOCK cells remained stable. The maximum fluorescence intensity of the screening round was observed in round 9 (Figure 1B). Therefore, the ssDNAs enriched in round 9 were selected for high‐throughput sequencing using Illumina MiSeq.

Based on abundance and homogeneity, the sequenced ssDNAs were classified into six families (Figure S2, Supporting Information). Eight representative sequences (YC1 to YC8) were synthesized as aptamer candidates for further evaluation. The binding ability of these aptamers was assessed by flow cytometry. Interestingly, all eight sequences showed specific binding to the TSHR‐293T cells but not to the MOCK cells. Among them, YC1, YC2, YC3, YC6, YC7, and YC8 exhibited relatively intense fluorescence signals (Figure 1C). Generally, ligand binding typically induces conformational changes in the TSHR, which can result in the activation or inhibition of multiple intracellular signaling pathways. Previous studies have shown that activating TSHR with M22, a human TSAb, increases intracellular cyclic adenosine monophosphate (cAMP) production and the phosphorylation of ERK1/2.^[^ ^12^, ^37^ ^]^ To further screen inhibitory aptamers, a specific functional selection process based on cAMP level and p‐ERK1/2 expression was employed. Notably, under non‐stimulated conditions, none of the aptamer candidates influenced cAMP levels in either MOCK cells or TSHR‐293T cells. In contrast, YC3 markedly inhibited cAMP production in M22‐stimulated TSHR‐293T cells (Figure 1D). Furthermore, YC3 effectively downregulated M22‐induced phosphorylation of ERK1/2 in TSHR‐293T cells (Figure 1E). Immunoblotting showed the inhibitory effect of YC3 on M22‐induced phosphorylation of both ERK1/2 and MEK in a dose‐dependent manner (Figure 1F,G). These findings collectively demonstrate that YC3 has significant potential to inhibit TSHR signaling. Based on its specific binding and inhibitory effects, YC3 was selected for further investigation.

### 2.2 Optimization, Characterization, and Modification of Aptamer YC3

**Figure 2:**
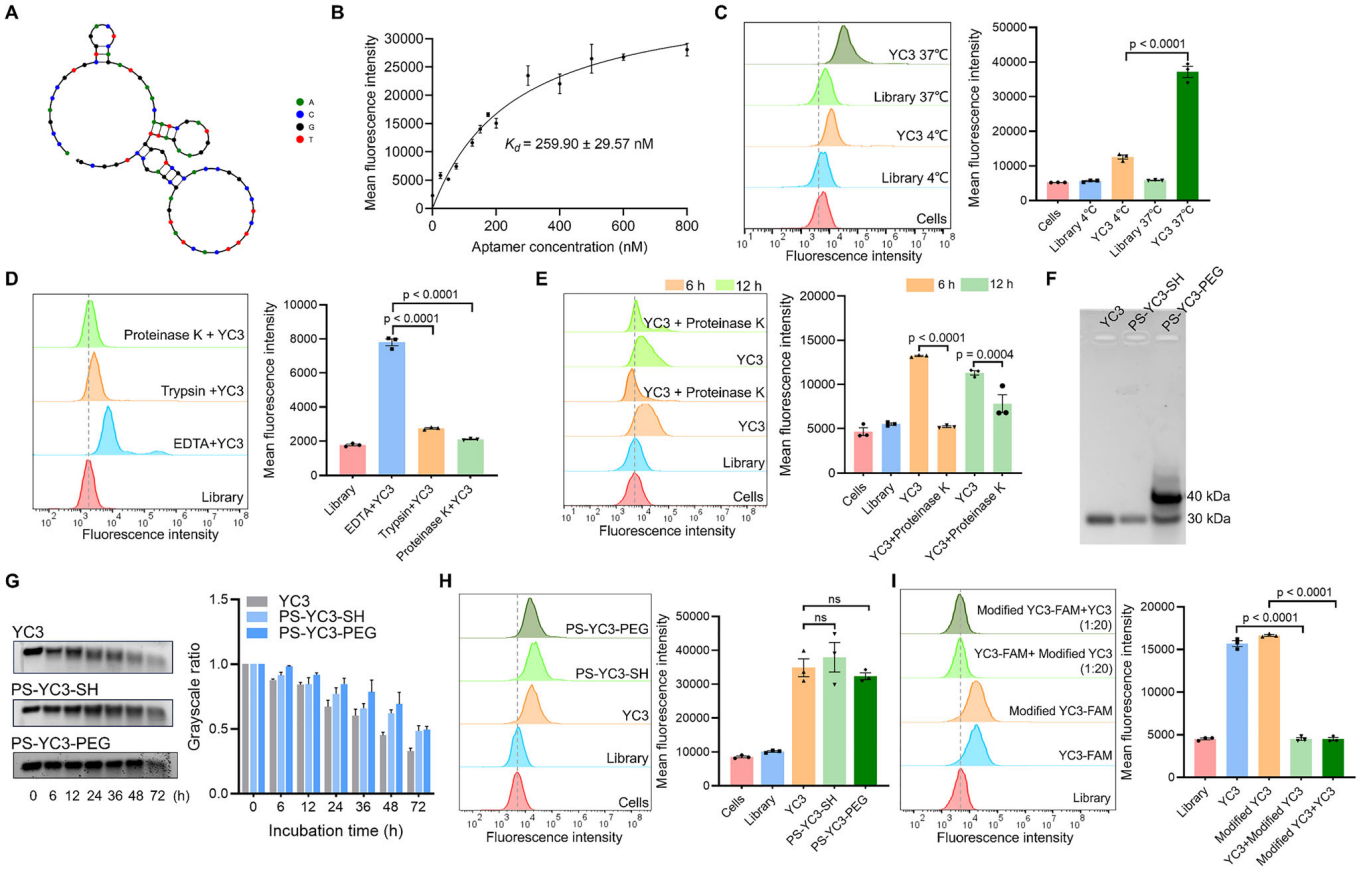
Characterization and modification of aptamer YC3. A) The secondary structure of YC3 was predicted by NUPACK software. B) The equilibrium dissociation constant of YC3 for TSHR‐293T cells was determined by flow cytometry (n = 3), using the following equation: Y = *B_max_* X/(*K_d_* +X) (X representing the concentration of aptamer, Y representing the mean fluorescence intensity, and *B_max_* representing the peak fluorescence intensity). C) Flow cytometry was employed to determine the binding ability of YC3 for TSHR‐293T cells at 4 or 37 °C. The FAM‐labeled initial Library was used as the control. Quantitative analysis of mean fluorescence intensity of YC3 (n = 3). D) Flow cytometry was employed to determine the binding ability of YC3 for TSHR‐293T cells treated with 0.25% trypsin or 10 µg mL^−1^ proteinase K. The TSHR‐293T cells treated with EDTA were used as the control. Quantitative analysis of mean fluorescence intensity of YC3 (n = 3). E) Flow cytometry was employed to determine the mean fluorescence intensity of TSHR‐293T cells after incubation with YC3 for 6 or 12 h, with or without treatment with 10 µg mL^−1^ proteinase K. Quantitative analysis of mean fluorescence intensity of YC3 (n = 3). F) Gel electrophoresis analysis of unmodified YC3 and its two modified forms. G) Agarose gel electrophoresis and densitometric quantitative analysis showing stability of YC3 and its modified forms in mouse serum (n = 3). H) Flow cytometry was employed to compare the binding ability between YC3 and its modified forms (n = 3). I) Flow cytometry was employed to analyze the competitive binding between YC3 and PS‐YC3‐PEG (Modified YC3) with indicated concentrations (n = 3). All data are represented as mean ± SEM. Two‐tailed unpaired Student's t‐tests were used to calculate P values. Accurate P values are listed in the figures.

In general, aptamers specifically bind to targets through unique 3D structures determined by sequences (Figure 2A). To further optimize the sequence of YC3, we designed two truncated schemes based on the predicted secondary structure using NUPACK (https://www.nupack.org/). The full‐length YC3 was truncated by removing the bases that formed the terminal stem‐loop structures (Figure S3A, Supporting Information). The resulting sequences are presented in Table S1 (Supporting Information). Flow cytometry showed that truncated YC3 sequences diminished binding ability to TSHR‐293T cells, highlighting the importance of the full YC3 structure for high affinity (Figure S3B, Supporting Information). Additionally, flow cytometry revealed that the equilibrium dissociation constant (*K_d_*) of YC3 against TSHR‐293T cells was 259.90 ± 29.57 nM (Figure 2B), indicating a strong binding affinity between YC3 and TSHR‐293T cells. It has been reported that the structures of aptamers can change slightly at different temperatures, which may affect their affinity.^[^ ^38^ ^]^ Since YC3 was generated using cell‐SELEX at 4 °C, we investigated its binding ability at physiological temperature. Incubation of YC3 with TSHR‐293T cells at 37 °C produced a stronger fluorescence signal compared to incubation at 4 °C (Figure 2C), indicating that physiological temperature enhances the binding affinity of YC3.

To assess the effect of cell membrane protein removal on YC3 binding, TSHR‐293T cells were treated with trypsin or proteinase K before incubation with YC3. Treatment with either enzyme significantly reduced YC3 binding to TSHR‐293T cells (Figure 2D), indicating that the targets of YC3 are located on the cell membrane, aligning with the known membrane location of TSHR. To assess the internalization of YC3, TSHR‐293T cells were incubated with FAM‐labeled YC3 for 6 and 12 h at 37 °C. Post‐incubation, proteinase K was applied to remove any YC3 remaining on the cell surface. Flow cytometry analysis revealed a significant reduction in fluorescence intensity after proteinase K treatment, indicating that YC3 did not internalize into TSHR‐293T cells within the 12‐h incubation period (Figure 2E).

Aptamers are widely known for their poor stability and high elimination rate in vivo. Therefore, enhancing the biological stability of aptamers is critical for both in vivo and in vitro applications. To address this, we modified YC3, resulting in PS‐YC3‐PEG, by substituting five standard oligonucleotides at both termini with phosphorothioate oligonucleotides, adding a sulfhydryl group to the 3' end, and subsequently performing PEGylation. After modification, the molecular weight of YC3 increased (Figure 2F), and its serum stability was enhanced without compromising its binding ability to TSHR‐293T cells (Figure 2G,H). We further investigated the binding sites of YC3 and PS‐YC3‐PEG through competitive binding analysis. As illustrated in Figure 2I, excess unlabeled PS‐YC3‐PEG inhibited the binding of FAM‐labeled YC3 to TSHR‐293T cells, and excess unlabeled YC3 similarly inhibited FAM‐labeled PS‐YC3‐PEG. This suggests that YC3 and PS‐YC3‐PEG share the same binding sites on TSHR‐293T cells.

### 2.3 Verification of TSHR as the Binding Target of YC3

**Figure 3:**
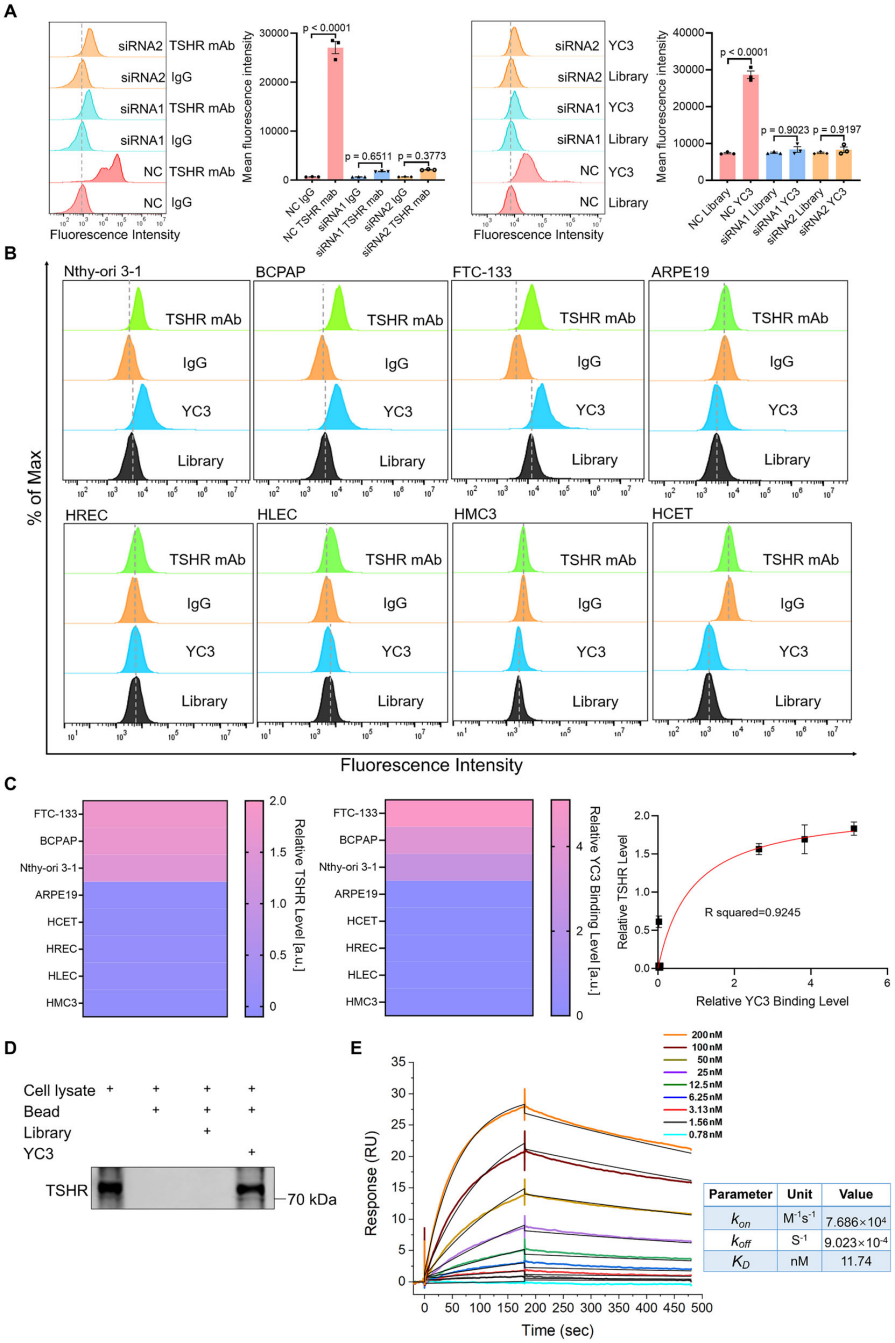
Verification of TSHR as the binding target of aptamer YC3. A) Flow cytometry analysis of TSHR expression levels using TSHR mAb, as well as the binding ability of YC3 to Nthy‐ori 3‐1 cells following transfection with TSHR siRNA1, TSHR siRNA2 or negative control siRNA (NC). Quantitative analysis of the relative fluorescence intensity (n = 3). B) Flow cytometry was employed to analyze TSHR protein expression levels using TSHR mAb, with IgG serving as a negative control, and evaluate the binding affinity of aptamer YC3, with the initial pool Library serving as a negative control, in various cell lines, including thyroid (Nthy‐ori 3‐1), thyroid carcinoma (BCPAP and FTC‐133), and ocular (ARPE19, HREC, HLEC, HMC3, and HCET) cell lines. C) Heatmap displaying the relative TSHR levels and YC3 binding levels in different cell lines (left panel), and correlation between the TSHR expression and the YC3 binding across these cell lines (right panel). Data represent three independent experiments (n = 3). D) Aptamer pull‐down assay. Lane 1: cell lysate; Lane 2: the proteins pulled down by naked beads; Lane 3: the proteins pulled down by aptamer Library‐conjugated beads; Lane 4: the proteins pulled down by aptamer YC3‐conjugated beads. Samples were stained with the anti‐TSHR antibody. E) A standard kinetics SPR assay demonstrated the binding of YC3 to immobilized TSHR with a *K_D_* of 11.74 nM, a *K_on_* of 7.686 × 10^4^ M^−1^s^−1^, and a *K_off_* of 9.023 × 10^−4^ s^−1^. All data are represented as mean ± SEM. One‐way ANOVA, followed by Tukey's multiple post hoc test, was used to calculate P values (A,B). Accurate P values are listed in the figures.

To confirm whether TSHR is the bona fide binding target of YC3, TSHR‐specific siRNAs were transfected into Nthy‐ori 3‐1 cells to knock down TSHR expression. Flow cytometry analysis revealed that TSHR silencing reduced TSHR expression and correspondingly decreased YC3 binding to Nthy‐ori 3‐1 cells (Figure 3A). To further investigate the relationship between YC3 binding and TSHR expression across different cell types, we analyzed membrane TSHR expression levels using TSHR‐specific antibodies. TSHR was expressed in the thyroid cell line (Nthy‐ori 3‐1) and thyroid cancer cell lines (BCPAP and FTC‐133), but was absent in ocular cell lines (ARPE19, HREC, HLEC, HMC3, and HECT). The YC3 binding strongly correlated with the membrane TSHR expression levels (Figure 3B), with a Pearson correlation coefficient of 0.9245 (Figure 3C). These findings demonstrate that YC3 exhibits high specificity, with binding levels strongly correlating with membrane TSHR expression across diverse cell lines. To further confirm the target of YC3, the membrane proteins of TSHR‐293T cells were extracted and subjected to an aptamer pull‐down assay. As shown in Figure 3D, TSHR was detected in the protein pulled down by the YC3 group but not in the control Bead group or Library group, demonstrating that YC3 binds to TSHR. Additionally, the binding affinity between the TSHR recombinant protein and the YC3 was determined by surface plasmon resonance (SPR), yielding an equilibrium dissociation constant (*K_D_*) of 11.74 nM, an association rate constant (*k_on_*) of 7.686×10^4^ M^−1^s^−1^, and a dissociation rate constant (*k_off_*) of 9.023×10^−4^ s^−1^ (Figure 3E). Taken together, these findings demonstrate that YC3 specifically binds to the TSHR protein with high affinity, further supporting its potential for TSHR‐targeting applications.

### 2.4 Aptamer YC3 Interacts with TSHR Through Allosteric Binding Sites

**Figure 4:**
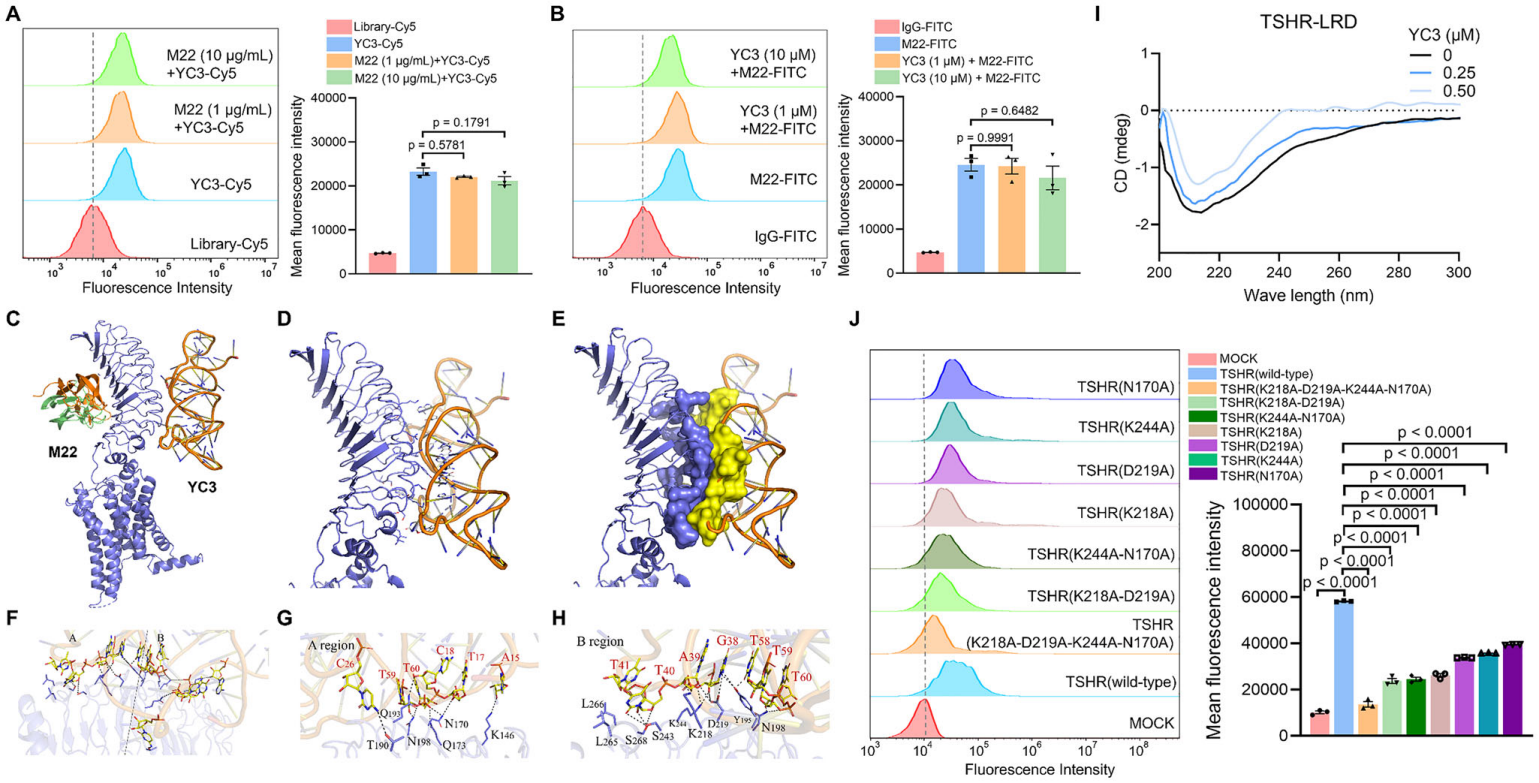
Aptamer YC3 interacts with TSHR through allosteric binding sites. A and B) Competitive binding assay of YC3 and M22 by flow cytometry. Quantitative analysis of the relative fluorescence intensity (n = 3). C) Molecular dynamic simulation results of binding sites at the interface between aptamer YC3, M22, and TSHR. D and E) Close view of TSHR and YC3 interaction. F–H) Two regions (A region shown in G and B region shown in H) of the binding sites between aptamer YC3 and TSHR were enlarged and represented as stick models. The key residues are marked in black. I) Circular dichroism spectra analysis for YC3‐mediated TSHR‐LRD conformational change. J) Flow cytometry results of binding between YC3 and HEK293T cells expressing wild‐type and mutant TSHR proteins. Quantitative analysis of the relative fluorescence intensity (n = 3). All data are represented as mean ± SEM. Two‐tailed unpaired Student's t‐tests (A,B) and one‐way ANOVA, followed by Tukey's multiple post hoc test (J), were used to calculate P values. Accurate P values are listed in the figures.

In humans, the mature full‐length TSHR protein is composed of an extracellular region, which includes a large leucine‐rich repeat domain (LRD) and a hinge region, a transmembrane domain, and an intracellular region.^[^ ^12^ ^]^ The TSHR‐LRD, rich in β‐sheet structure, is the primary site for thyrotropin and autoantibodies binding. These ligands interact with amino acids located in the concave surface of the domain.^[^ ^10^, ^39^, ^40^ ^]^ To investigate the binding sites between the aptamer YC3 and TSHR, we conducted a series of experiments to characterize the detailed interaction region. Initially, we performed a competitive binding analysis to determine if YC3 competes with M22 for TSHR binding. Flow cytometry showed that neither excess M22 nor YC3 inhibited the other's binding (Figure 4 A,B), which indicates that YC3 and M22 bind to different sites on the TSHR. To define the specific interaction region, we performed molecular dynamics simulations to map the YC3‐binding residues on TSHR. In a blind docking process, 100 conformations were generated, clustered, and ranked according to their docking energy values. The 50 conformations with the highest number of clusters and the lowest binding energies were selected to predict the protein‐aptamer binding interface. Analysis of these conformations revealed that, unlike M22, YC3 bound to the convex surface of the TSHR‐LRD (Figure S4A, Supporting Information). The complex structure with the lowest binding score from the precise docking is presented in Figure 4C–E. To characterize the dynamic behavior of the TSHR‐YC3 complex, a 100‐ns molecular dynamics simulation was performed, and the refined binding model was extracted from the MD trajectory. The binding interface is primarily located in two regions (Figure 4F), designated A and B, with the interaction dominated by a robust hydrogen bond network (Figure S4B, Supporting Information). Key residues of TSHR that form important interactions with YC3 include T190, Q193, Q173, N170, K146, L265, L266, S268, S243, K244, K218, D219, Y195, and N198 (Figure 4G,H). Residue binding free energy decomposition analysis revealed that K218, D219, K244, and N170 made the largest contributions to the binding energy of the complex in both the docking stage and the kinetic simulation (Table S2, Supporting Information).

To validate computationally predicted binding residues, we performed site‐directed mutagenesis on four key residues, mutating them to Alanine in quadruple, double, and single mutants. Flow cytometry assessed YC3 binding to HEK293T cells expressing these TSHR mutations. TSHR expression levels were comparable across all transfected cell types (Figure S5, Supporting Information). YC3 binding was most significantly reduced in cells with quadruple mutants, followed by double mutants, and then single mutants. The K218A single mutant caused the most substantial decrease in YC3 binding, highlighting its critical role (Figure 4J). These findings support the molecular dynamics simulations, confirming the importance of these residues for YC3 binding. To experimentally investigate the modeled conformational changes in TSHR‐LRD, we performed circular dichroism (CD) spectroscopy. The CD spectrum of TSHR‐LRD displays a pronounced negative ellipticity near 215 nm, consistent with a β‐sheet‐rich secondary structure, as previously reported for this domain.^[^ ^39^ ^]^ Upon titration of TSHR‐LRD with increasing concentrations of YC3, a progressive decrease in molar ellipticity at ≈215 nm was observed (Figure 4I), indicating a concentration‐dependent reduction in β‐sheet content. This evidence confirms that YC3 binding triggers conformational rearrangements in TSHR‐LRD, supporting our proposed allosteric regulatory mechanism. In summary, our results confirm YC3 as an allosteric regulator of TSHR and reveal its distinct binding epitope on the convex surface of the LRD.

### 2.5 Characterization of YC3 Targeting and In Vivo Properties

**Figure 5:**
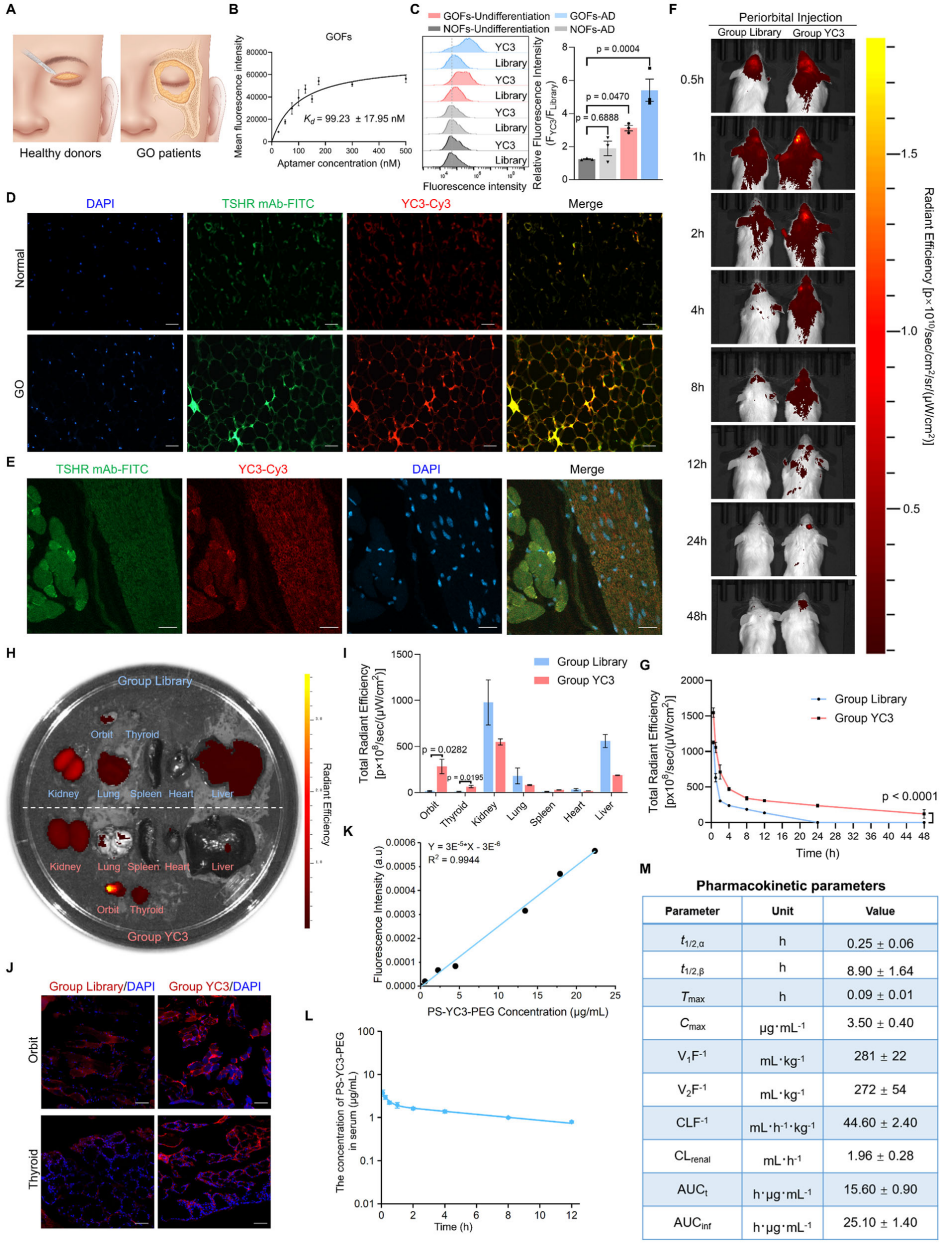
Characterization of YC3 targeting and in vivo properties. A) Schematic illustration of orbital adipose tissues from GO patients who underwent orbital decompression surgery, and healthy donors who underwent blepharoplasty. B) The equilibrium dissociation constant of YC3 for GOFs was determined by flow cytometry (n = 3), using the following equation: Y = *B_max_* X/(*K_d_* +X) (X representing the concentration of aptamer, Y representing the mean fluorescence intensity, and *B_max_* representing the peak fluorescence intensity. C) Flow cytometry analysis of the binding of YC3 to GOFs and normal NOFs with or without adipogenic differentiation (AD). Quantitative analysis of the relative fluorescence intensity (n = 3). D) Confocal microscopy was used to analyze the colocalization of anti‐TSHR antibody and YC3 on orbital adipose tissue sections from healthy donors and GO patients. Representative images were shown. Scale bars: 50 µm. E) Confocal microscopy was used to analyze the colocalization of anti‐TSHR antibody and YC3 on orbital tissue sections from BALB/c mice. Representative images were shown. Scale bars: 20 µm. F) In vivo imaging of aptamers in BALB/c mice after periorbital injection of Cy5‐labeled aptamers. The fluorescence of aptamers in mice was collected by the IVIS Lumina II imaging system at the indicated time. G) Quantitative analysis of total radiant efficiency showing Cy5‐labeled aptamers retention in the orbits corresponding to panel (F) (n = 3). H) Ex vivo fluorescence imaging of dissected orbits, thyroids, and major organs (kidney, lung, spleen, heart, liver) in BALB/c mice at 2 h post‐periorbital injection of Cy5‐labeled aptamers. I) Corresponding total radiant efficiency of dissected tissues in panel (H) (n = 3). The fluorescence signal was collected using the IVIS Lumina II imaging system. J) Frozen section fluorescence imaging of Cy5‐labeled aptamers in the corresponding dissected orbits and thyroids. The sections were counterstained with DAPI. Representative images were shown. Scale bars: 20 µm. K) A standard curve of Cy5‐labeled PS‐YC3‐PEG concentration (µg/mL) versus fluorescence intensity (a.u.) was generated for quantitative analysis. L and M) Pharmacokinetic profiles of PS‐YC3‐PEG showing blood concentration‐time curves and calculated pharmacokinetic parameters (n = 3). Parameters were determined using WinNonlin PK software employing a two‐compartment pharmacokinetic model. All data are represented as mean ± SEM. One‐way ANOVA, followed by Tukey's multiple post hoc test (C), and two‐way ANOVA (G) were used to calculate P values. Accurate P values are listed in the figures.

TSHR is considered the primary autoantigen in GO, and orbital fibroblasts (OFs) are the key effector cells and targets of autoimmune attacks.^[^ ^4^ ^]^ In GO development, enhanced TSHR expression is driven by autoimmune and inflammatory processes, paralleling de novo adipogenesis.^[^ ^11^ ^]^ Compared to healthy donors, TSHR expression was significantly higher in orbital tissues from GO patients (Figure S6A,B, Supporting Information). We then investigated YC3 binding in orbital cells and tissues. Graves' orbital fibroblasts (GOFs) and normal orbital fibroblasts (NOFs) were isolated from GO patients and healthy donors, respectively, and identified using the fibroblast marker Vimentin (Figure S6C, Supporting Information). YC3 showed strong binding affinity to GOFs, with a *K_d_* of 99.23 ± 17.95 nM (Figure 5B). Oil Red O staining revealed that fibroblasts with adipogenic differentiation became rounded and accumulated red‐stained lipid droplets (Figure S6D, Supporting Information). Notably, adipogenic differentiation significantly increased TSHR expression in GOFs but not in NOFs (Figure S6E,F, Supporting Information). Given these differences, we examined YC3 binding to GOFs and NOFs in both undifferentiated and adipogenic states. YC3 binding was stronger in GOFs than in NOFs, and even stronger in adipogenic GOFs (Figure 5C).

To determine whether the specific binding of YC3 to TSHR can be used as a molecular probe for clinical application, orbital adipose tissues from GO patients and healthy donors were incubated with Cy3‐labeled YC3 and FITC‐labeled TSHR antibodies. Compared to healthy donors, orbital adipose tissues from GO patients showed a higher expression of TSHR, as well as a stronger binding of YC3 (Figure 5D). Meanwhile, YC3 and TSHR showed strong colocalization in orbital adipose tissues. Moreover, we found that TSHR was expressed in mouse orbital tissues, with TSHR expression in the thyroid serving as a positive control (Figure S7A,B, Supporting Information). Strong colocalization of YC3 and TSHR was also observed in the orbital tissues of mice (Figure 5E). To investigate the targeting ability and biodistribution of YC3 in vivo, we performed periorbital injections in GO mice using equal amounts of Cy5‐labeled modified YC3 or Cy5‐labeled modified Library. Following injection, we observed that YC3 accumulated around the orbits of the mice and remained detectable at the site for an extended period, although the signal gradually diminished over time. In contrast, the Library did not show a significant accumulation around the orbits. Notably, at 24 h post‐injection, YC3 was still enriched in the periorbital region, whereas no such enrichment was observed for Library (Figure 5F,G). At 2 h post‐periorbital injection of Cy5‐labeled aptamers, YC3 exhibited specific accumulation in both the orbits and thyroids, whereas Library predominantly accumulated in the kidney, liver, and lung (Figure 5H–I). Fluorescence imaging of frozen tissue sections from dissected orbit and thyroid tissues confirmed that YC3 was accumulated in these target tissues (Figure 5J). Taken together, YC3 exhibits strong targeting specificity in both human and mouse orbit, highlighting its promising potential as a molecular probe for in vivo applications.

To systematically evaluate the pharmacokinetic profile of modified YC3, we conducted comprehensive studies in mice. A single periorbital injection of modified YC3 was administered, and the pharmacokinetic profile of the aptamer was monitored via Cy5 fluorescence. Pharmacokinetic analysis revealed favorable blood circulation characteristics, with distribution (t_1/2, α_) and elimination (t_1/2, β_) half‐lives of 0.25 ± 0.06 h and 8.90 ± 1.64 h, respectively. Notably, modified YC3 demonstrated superior clearance properties, with an apparent clearance (CLF^−1^) of 44.60 ± 2.40 mL·h^−1^·kg^−1^ and renal clearance (CL_renal_) of 1.96 ± 0.28 mL·h^−1^. These values are superior to the previously reported aptamer.^[^ ^41^, ^42^ ^]^ The blood concentration‐time profile of modified YC3, along with other key pharmacokinetic parameters, including the maximum concentration (C_max_), time to reach maximum concentration (T_max_), apparent volume of distribution in the central compartment divided by bioavailability (V_1_F^−1^), apparent volume of distribution in the peripheral compartment divided by bioavailability (V_2_F^−1^) and the area under the blood concentration‐time curve (AUC_t_ and AUC0_inf_), is depicted in Figure 5L,M. Collectively, modified YC3 demonstrates favorable pharmacokinetic properties, supporting its potential for further development.

### 2.6 YC3 Attenuates Disease‐associated Phenotypes in Orbital Fibroblasts

In GO, dysregulated activity of OFs results in pathological tissue remodeling, which is marked by inflammation, edema, fibrosis, and increased adipogenesis. GO is traditionally classified as either active or inactive, following the trajectory described by Rundle's curve, with inflammation predominating in the active phase and fibrosis characterizing the inactive phase.^[^ ^43^ ^]^ Both TSH and M22 significantly increased IL‐6 and IL‐8 mRNA and protein levels in GOFs. M22, but not TSH, also elevated IL‐1β mRNA and protein levels (Figure S8A,B, Supporting Information). Hyaluronan (HA) is a key component of the extracellular matrix (ECM), and its dysregulation is implicated in inflammatory and fibrotic diseases.^[^ ^44^ ^]^ Therefore, we also examined the effects of TSH and M22 on hyaluronan synthases (HAS1, HAS2, and HAS3) in GOFs. The results showed that M22, but not TSH, significantly increased the mRNA levels of these synthetases and HA levels (Figure S8C,D, Supporting Information).

**Figure 6:**
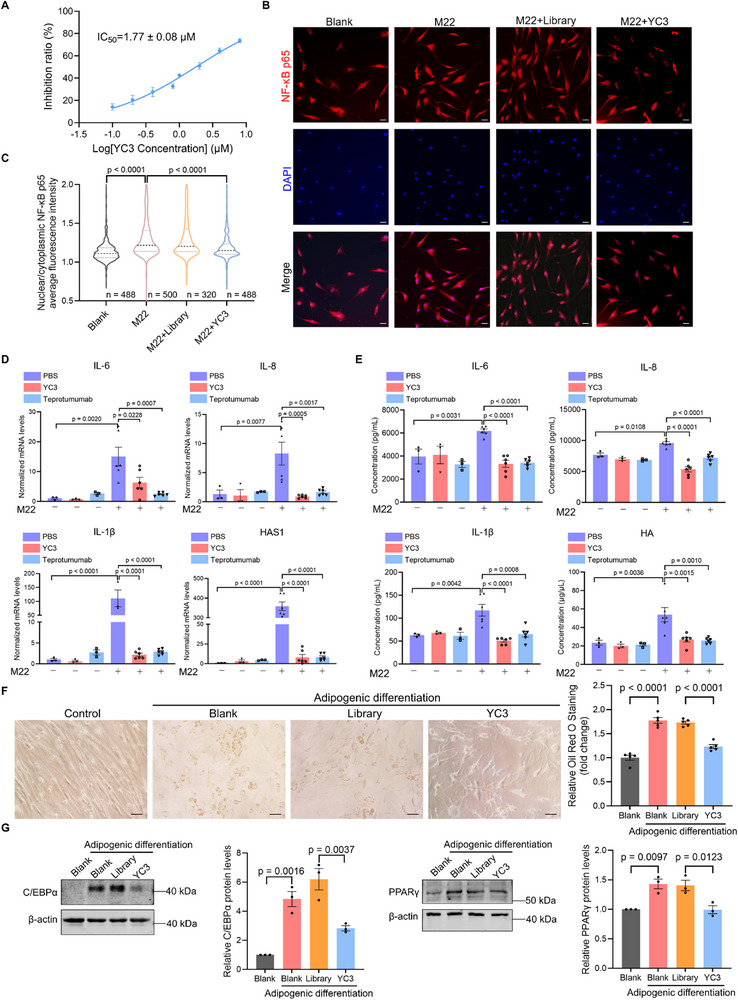
YC3 attenuates disease‐associated phenotypes in orbital fibroblasts. A) cAMP production assay in GOFs stimulated with M22 and treated with indicated concentrations of YC3 (n = 4). Data are presented with half‐maximal inhibitory concentration (IC_50_) values. B) High‐content imaging analysis of NF‐κB p65 in GOFs stimulated with M22 and treated with either an aptamer Library or YC3 (2 µM). Representative images are shown. Scale bars: 50 µm. C) Violin plots showing the nuclear/cytoplasmic ratio of NF‐κB p65 fluorescence intensity, as determined by the high‐content imaging system in panel (B). D) The mRNA expression levels of IL‐6, IL‐8, IL‐1β, and HAS1 in GOFs stimulated by M22 and treated with aptamer YC3 (2 µM) or Teprotumumab (5 µg mL^−1^) by qPCR, n = 3 independent samples in the non‐stimulated group, n = 6 independent samples in the M22‐stimulated group. E) The concentrations of IL‐6, IL‐8, IL‐1β, and HA in the supernatant medium of GOFs stimulated by M22 and treated with either aptamer YC3 (2 µM) or Teprotumumab (5 µg mL^−1^) were measured by ELISA, n = 3 independent samples in the non‐stimulated group, n = 6 independent samples in the M22‐stimulated group. F) Representative images of Oil Red O staining in GOFs treated with either aptamer Library or YC3 (2 µM) after adipogenic differentiation. Scale bars: 50 µm. Quantitative analysis of the relative Oil Red O staining values of cells (n = 5). G) Representative immunoblots/densitometric quantitative analysis of C/EBPα and PPARγ protein levels in GOFs after adipogenic differentiation and treatment with aptamer Library or YC3 (2 µM). β‐actin was used as a loading control (n = 3). All data are represented as mean ± SEM. One‐way ANOVA, followed by Tukey's multiple post hoc test, was used to calculate P values. Accurate P values are listed in the figures.

To further investigate the effects of YC3 on GOFs, we examined several key cellular processes. As the primary second messenger of TSHR signaling, cAMP plays a central role in downstream signal transduction. YC3 significantly inhibited M22‐stimulated cAMP production in GOFs, with an IC_50_ of 1.77 µM (Figure 6A). Inflammation predominates during the active phase of GO, and the nuclear translocation of NF‐κB p65 is a critical step in the inflammatory response. To simulate the characteristics of active‐phase GOFs in vitro, M22 was used to stimulate GOFs. Notably, YC3 attenuated M22‐induced nuclear translocation of NF‐κB p65 (Figures 6B,C; Figure S9, Supporting Information). We further assessed the effect of YC3 on inflammatory cytokine production and HA synthesis. Consistent with its anti‐inflammatory potential, YC3 significantly reduced both mRNA and protein levels of IL‐6, IL‐8, and IL‐1β in M22‐stimulated GOFs. Moreover, YC3 downregulated the expression of HAS1 mRNA and decreased HA production. The anti‐inflammatory and anti‐ECM production effects of YC3 were comparable to those of the clinical drug Teprotumumab (Figure 6D,E). Moreover, fibroblast activation enhances contractility and migration, which is largely driven by actin‐myosin interactions and the formation of stress fibers.^[^ ^45^ ^]^ Our results also demonstrate that YC3 significantly reduces M22‐induced actin fiber density and stress fiber formation in GOFs (Figure S10, Supporting Information).

Adipogenesis is another core pathological feature of GO, and the differentiation of OFs into adipocytes represents a key cellular process. Therefore, the effect of YC3 on the adipogenic differentiation of GOFs was evaluated. Adipogenic differentiation was characterized by distinct morphological changes, including cell rounding and the formation of lipid droplets. Compared to Library‐treated controls, YC3‐treated GOFs exhibited reduced cell rounding and decreased lipid accumulation (Figure 6F). Consistent with these morphological changes, YC3 significantly downregulated protein levels of key adipogenic markers, including CCAAT/enhancer‐binding protein alpha (C/EBPα) and peroxisome proliferator‐activated receptor gamma (PPARγ) (Figure 6G). These findings demonstrate that YC3 effectively suppresses the adipogenic differentiation of GOFs. Collectively, YC3 decreases the secretion of inflammatory factors, HA synthesis, and adipogenesis, thereby mitigating GO disease‐associated phenotypes in orbital fibroblasts.

### 2.7 Aptamer YC3 Improves Outcomes in the Graves' Ophthalmopathy Mouse Model

The GO mouse model was established following a previous report.^[^ ^29^ ^]^ At week 18, serum levels of T3, T4, and thyrotropin receptor antibody (TRAb) in mice from the GO group were significantly elevated compared to those in the MOCK group (Figure S11A, Supporting Information). For ocular symptoms, mice from the GO group exhibited signs of inflammation, including eyelid broadening, eyelid swelling, and congestion, which resembled the symptoms observed in GO patients (Figure S11B, Supporting Information). To monitor inflammatory events and remodeling of orbital tissues in vivo, we performed serial non‐invasive Magnetic Resonance Imaging (MRI) on living mice. T2‐weighted MRI was used to distinguish orbital tissues, including extraocular muscle, adipose tissue, optic nerve, and anatomical structures. The MRI images from the GO group showed extraocular muscle hypertrophy and increased signal intensity, indicating pathological changes such as inflammation and edema in the orbital tissues (Figure S11C, Supporting Information). These findings are similar to the features observed in GO patients.

**Figure 7:**
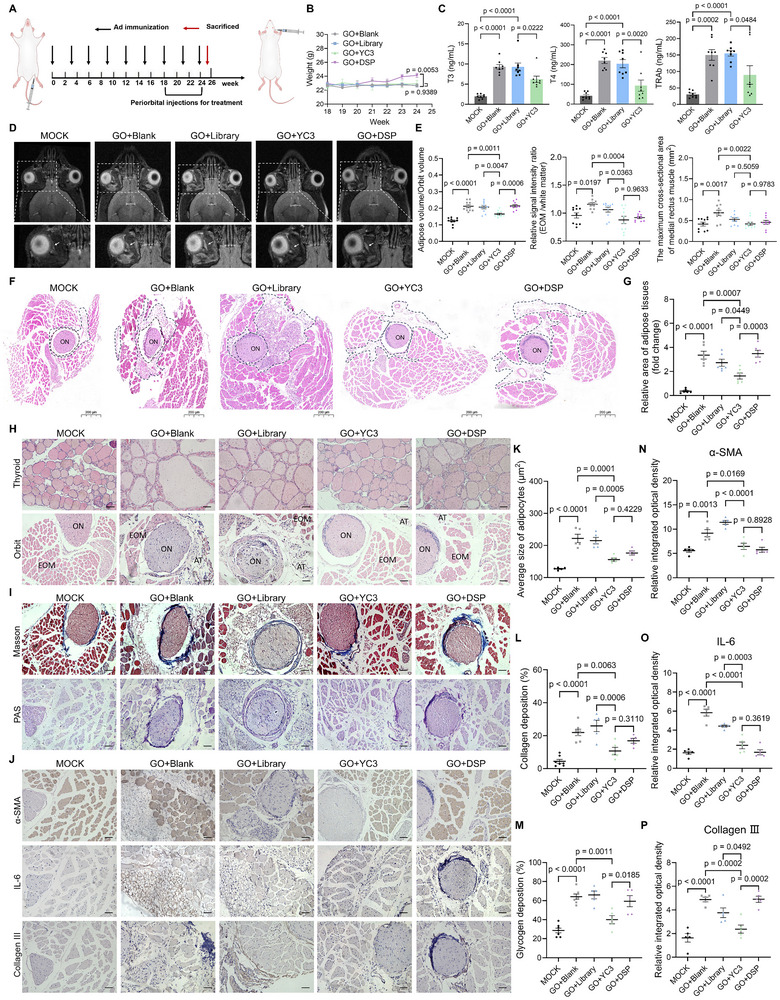
Aptamer YC3 improves outcomes in the GO mouse model. A) Experimental timeline for model construction and treatment of mice. Mice were injected with Ad‐TSHR289 or Ad‐MOCK, and then mice injected with Ad‐TSHR289 were randomly grouped, followed by treatment with PBS, Library, YC3, or DSP, respectively. B) Body weight changes in four treatment groups were measured weekly during the treatment period. C) The concentrations of T3, T4, and TRAb in mice serum in four groups were measured by ELSIA (n = 8). D) Representative T2‐weighted Magnetic Resonance Imaging (MRI) of the mouse orbits from the five mouse groups. The lower images provide an enlarged view of the areas within the white dashed boxes shown in the corresponding upper images. White arrows denote the extraocular muscle. E) Quantitative analysis of MRI images from the five mouse groups (n = 10). EOM, extraocular muscle. F) Representative Hematoxylin‐eosin (H&E) staining images of orbital tissue sections from the five mouse groups. ON, optic nerve. The region outlined by the black dashed line represents adipose tissue. Scale bars: 200 µm. G) Quantification of adipose tissue area relative to the optic nerve area in orbital tissue sections from the five mouse groups (n = 6), corresponding to panel (F). H) Representative H&E staining images of thyroidal and orbital tissue sections from the five mouse groups. ON, optic nerve; EOM, extraocular muscle; AT, adipose tissue. Scale bars: 20 µm. I) Representative Masson staining images and periodic acid‐schiff (PAS) staining images of orbital tissue sections from the five mouse groups. Scale bars: 20 µm. J) Representative immunostaining images of α‐SMA, IL‐6, and Collagen III in orbital tissue sections from the five mouse groups. Scale bars: 20 µm. K) Quantification of the average size of adipocytes in orbital tissue sections from the five mouse groups (n = 5). L and M) Statistical analysis of Masson staining and PAS staining from orbital tissue sections in the MOCK group (n = 7), the GO+Blank group (n = 7), the GO+Library group (n = 5), the GO+YC3 group (n = 5), and the GO+DSP group (n = 5), corresponding to panel (I). N‐P) Statistical analysis of IHC images by calculating the relative integrated optical density (IOD) (n = 5), corresponding to panel (J). All data are represented as mean ± SEM. Two‐way ANOVA, followed by Dunnett's multiple comparisons test (B) and one‐way ANOVA, followed by Tukey's multiple post hoc test (C,E,G,K–P), were used to calculate P values. Accurate P values are listed in the figures.

As shown in the timeline (Figure 7A), after seven injections of the adenovirus, mice with GO were randomly divided into four groups: periorbital injection with PBS (GO+Blank group); periorbital injection with the modified aptamer Library (GO+Library group); periorbital injection with the modified aptamer YC3 (GO+YC3 group); periorbital injection with dexamethasone sodium phosphate (DSP) (GO+DSP group), serving as a positive control. During the treatment, the weight of mice remained stable, except for those in the GO+DSP group, which gained weight during the treatment (Figure 7B). At week 24, the end of treatment, serum levels of T3, T4, and TRAb in mice from the GO+YC3 group were significantly lower than those in the GO+Library group (Figure 7C). In T2‐weighted MRI images, extraocular muscle hypertrophy, orbital adipogenesis, and orbital tissue inflammation and swelling were more pronounced in the GO+Blank and GO+Library groups compared to the GO+YC3 group (Figure 7D,E). These findings indicate that YC3 alleviates the pathological alterations of orbital tissues.

In the histopathological analysis, orbital sections from the GO+Blank group exhibited inflammatory cell infiltration and expanded orbital adipose tissue. Interestingly, YC3 ameliorated the morphological and histological alterations in the orbit, reducing inflammatory cell infiltration and attenuating adipose tissue expansion (Figure 7F–H,K). Thyroid sections from the GO+Blank group revealed diffuse enlargement of the thyroid gland. The thyroid follicles appeared cuboidal to cylindrical with a small amount of colloid, indicating thyroid hyperactivity (Figure 7H). Fibrosis can be caused by excessive and abnormal accumulation of ECM, which can impair tissue function.^[^ ^46^, ^47^ ^]^ Mice in the MOCK group exhibited minimal collagen and glycogen deposition in the orbital tissues. In contrast, the GO+Blank and GO+Library groups showed a significant increase in collagen and glycogen accumulation around the optic nerve and extraocular muscle bundles. Notably, YC3 ameliorated the accumulation of collagen and glycogen, thereby mitigating the fibrotic response in orbits (Figure 7I,L,M). We further examined the expression levels of fibrosis markers, alpha‐smooth muscle actin (α‐SMA) and collagen III, as well as the inflammatory cytokine IL‐6, in the orbital tissues. Our findings demonstrated that in the GO+Blank group and the GO+Library group, the levels of α‐SMA, IL‐6, and collagen III increased. In contrast, YC3 treatment significantly reduced the expression of these markers (Figure 7J,N–P). In addition, potential systemic toxicity was assessed via H&E staining of major organs, with results showing no pathological alterations after YC3 treatment (Figure S12, Supporting Information). Taken together, these results demonstrate that YC3 significantly improves disease outcomes in GO mice by alleviating hyperthyroidism, reducing inflammation, decreasing ECM accumulation, inhibiting adipogenesis, and mitigating tissue fibrosis, with a favorable short‐term safety profile.

## 3 Discussion

The TSHR inhibition represents a compelling strategy to disrupt the pathogenic cascade of GO. The diagnostic and staging value of TSHR is supported by evaluating the percentages of TSHR‐positive cells in the peripheral blood of GO patients.^[^ ^48^ ^]^ The therapeutic value of TSHR inhibition is supported by phase I clinical trial data demonstrating that the TSHR monoclonal antibody K1‐70 reduces exophthalmos in GO patients.^[^ ^49^ ^]^ Nucleic acid aptamers, often termed “chemical antibodies”, are functionally comparable to traditional antibodies but offer distinct advantages, including smaller size, rapid chemical synthesis, versatile chemical modification, relative high stability, and lack of immunogenicity.^[^ ^50^ ^]^ Building on this foundation, YC3 was developed as the first TSHR‐targeting aptamer with inhibitory activity. YC3 functions dually as a molecular probe for TSHR mapping and a therapeutic inhibitor. Preclinical evaluation of YC3 includes both in vitro and in vivo studies, with in vivo analyses comprising imaging examinations and histological assessments of orbital tissues. These investigations provide robust mechanistic insights and pharmacodynamic investigation of TSHR inhibition in GO mouse model. GO progresses through active and inactive stages.^[^ ^43^ ^]^ Given that fibrotic orbital remodeling during the inactive phase is often intractable, early intervention during the active stage is crucial. In OFs, YC3 reduces inflammation, HA synthesis, and adipogenesis, thereby mitigating key pathological features of GO. These effects position YC3 as a particularly suitable therapeutic candidate for active GO. Moreover, YC3 administration not only improved ocular pathology but also alleviated hyperthyroidism in the GO mice. This dual therapeutic benefit, consistent with its mechanism, underscores YC3's potential to address both the ocular and systemic manifestations of Graves' disease. Collectively, through strong pharmacodynamic activity and mechanistic elucidation, YC3 provides preclinical evidence that TSHR inhibition ameliorates GO pathogenesis, establishing it as a promising therapeutic candidate.

Among the reported TSHR inhibitors, both orthosteric and allosteric inhibitors have been identified. The orthosteric inhibitor K1‐70 binds to the same binding sites on the TSHR as TSH and TSAbs, thereby preventing ligand binding and receptor activation.^[^ ^51^ ^]^ In contrast, current allosteric TSHR inhibitors are primarily small‐molecule compounds that target the transmembrane domain or extracellular loops of the receptor, yet their binding mechanisms remain poorly characterized. For example, the mechanisms of the allosteric inhibitors Org41841 and VA‐K‐14 have been investigated using molecular docking approaches.^[^ ^52^, ^53^ ^]^ The results indicate that allosteric inhibitors may target a pocket formed by the seven transmembrane helices and three extracellular loops of the TSHR, maintaining the receptor in an inactive conformation.^[^ ^12^ ^]^ These allosteric inhibitors block signal transduction from the extracellular to the transmembrane region, thereby preventing the conformational changes required for TSHR activation. However, the proposed allosteric inhibitory mechanisms remain experimentally unvalidated, and the potential involvement of other allosteric sites in TSHR activation remains undetermined. In this study, we systematically investigated the structural and functional basis of the YC3‐TSHR interaction. Employing an integrative approach combining competition binding assays, computational structural modeling, and site‐directed mutagenesis, we mapped YC3's binding interface on TSHR and confirmed its allosteric inhibitory role. Intriguingly, YC3 binding does not compete with TSAbs, which occupy the concave face of the LRD in TSHR's extracellular domain. Instead, computational structural modeling and experimental data indicate that YC3 binds to the convex face of the LRD, a previously uncharacterized allosteric site. Notably, the YC3‐TSHR interaction interface is extensive. The mutagenesis analyses revealed that mutations at key residues modestly reduced YC3's binding affinity without completely abolishing it, suggesting multi‐site interactions across the YC3‐TSHR interface. This broad interaction interface likely underpins YC3's nanomolar affinity and high specificity. Among these key residues, the K218 mutation most significantly compromises the binding ability of YC3, demonstrating its critical role. However, the functional role of these residuals in TSHR activation remains enigmatic and warrants further investigation. Structural analysis further revealed that YC3 binding induces a reduction in the β‐sheet content of the TSHR‐LRD, indicating significant conformational rearrangements in this functionally essential domain. These changes may destabilize the active conformation of the receptor, thereby impairing downstream signaling pathways. Our findings show that YC3 exerts its inhibitory effect via a novel allosteric mechanism, distinct from both orthosteric antibodies and previously proposed small‐molecule allosteric inhibitors. Future structural biology studies, particularly high‐resolution structural determination of the YC3‐TSHR complex, will further validate this allosteric mechanism and deepen mechanistic understanding of YC3. Our findings reveal a multifaceted regulatory mechanism governing TSHR signaling, highlighting the intricate interplay between ligand binding and conformational dynamics. Crucially, this study has identified a previously unidentified allosteric site on the convex surface of the TSHR‐LRD by elucidating the structural and functional basis of the YC3 mechanism. Spatially and mechanistically distinct from the classical orthosteric ligand‐binding domain, these insights open new avenues for the rational design of next‐generation allosteric inhibitors.

Aptamer‐based inhibitors have gained significant attention and spurred active development due to their advantages and clinical success. Most aptamers exert their inhibitory effects through a competitive mechanism, wherein they bind directly to the receptor's orthosteric site, thereby blocking interactions with endogenous ligands and modulating downstream signaling pathways.^[^ ^36^, ^54^, ^55^, ^56^ ^]^ However, this competitive mode of action presents certain limitations. In particular, its efficacy can be compromised under conditions of high ligand concentration, and it may lead to reduced selectivity and unintended off‐target effects, potentially resulting in adverse events.^[^ ^57^ ^]^ In contrast, allosteric modulators offer a promising alternative by engaging with regions of the target that are spatially and mechanistically distinct from the orthosteric binding site. This non‐competitive mechanism allows for enhanced therapeutic precision, as allosteric modulation can achieve robust efficacy at lower doses while minimizing interference with native ligand‐receptor interactions. YC3 represents a paradigm shift in aptamer‐based therapeutics by functioning as a non‐competitive, allosteric inhibitor of TSHR. Unlike conventional aptamers, YC3 does not compete with endogenous ligands. This allosteric mechanism expands the functional repertoire of aptamers beyond competitive blockade, opening new avenues for the development of highly specific and efficacious allosteric aptamers and unlocking new therapeutic possibilities against previously intractable targets.

The single‐stranded nucleic acid structure of aptamers renders them inherently susceptible to nuclease‐mediated degradation in biological environments, while their relatively low molecular weight predisposes them to rapid renal clearance. These pharmacokinetic limitations pose significant challenges for clinical applications, where frequent dosing regimens may be clinically suboptimal and impractical. In this context, local administration offers a promising strategy by enabling targeted delivery to specific tissues, minimizing systemic exposure, and achieving substantially higher local drug concentrations, which are particularly advantageous for aptamer‐based therapeutics. Notable examples of FDA‐approved aptamer therapies include Pegaptanib and Zimura, both of which are administered via intravitreal injection.^[^ ^36^ ^]^ These agents exemplify the successful application of local administration to maximize therapeutic efficacy while minimizing systemic exposure. Based on this, YC3 was chemically modified to enhance its stability and increase molecular weight while preserving its binding affinity. Furthermore, periorbital injection was employed and yielded promising therapeutic efficacy. To advance YC3 toward therapeutic application, further iterative optimization will be essential. This includes refining chemical modifications to maximize stability, conducting comprehensive pharmacodynamic and toxicological assessments, and mapping concentration gradients to define the therapeutic window. Such studies will be essential to ensure sustained therapeutic efficacy and long‐term systemic safety, and are crucial for translational development. These findings highlight the potential of YC3 as a next‐generation aptamer‐based therapeutic and underscore the broad applicability of aptamers in treating complex autoimmune diseases.

In summary, this study developed YC3, a TSHR‐specific nucleic acid aptamer, demonstrating inhibitory pharmacodynamic activity both in vitro and in vivo. Mechanistically, YC3 binds to the convex surface of the TSHR‐LRD, allosterically inhibiting receptor activation. These findings establish YC3 as a promising therapeutic candidate for GO and TSHR‐related diseases, identify a novel allosteric site for next‐generation inhibitors, and highlight the value of aptamers in uncovering receptor mechanisms and identifying previously cryptic druggable sites.

## 4 Experimental Section

### Materials

Yeast tRNA was purchased from Sigma‐Aldrich (Shanghai, China). Recombinant human TSH alpha/beta heterodimer protein was purchased from the R&D System (Minneapolis, USA). Human monoclonal autoantibody to the TSHR (M22) was purchased from RSR (Cardiff, UK). Teprotumumab was purchased from AntibodySystem (Strasbourg, France). All other reagents were purchased from Beyotime (Shanghai, China) unless stated otherwise.

### Ethical Statement

Human tissue specimens were obtained with approval from the Eye Hospital of Wenzhou Medical University Ethics Committee (Approval No. 2023‐074‐K‐62), and signed informed consent was obtained from all participants. All animal experiments were conducted in accordance with the guidelines of the Eye Hospital of Wenzhou Medical University and approved by the Institutional Animal Care and Use Committee (Approval No. YSG23110303).

### Human Orbital Fibroblast Isolation and Culture

Orbital adipose tissues were obtained from six patients with GO who underwent orbital decompression surgery for severe GO, and six healthy donors who underwent blepharoplasty. None of the GO patients had received steroid therapy or radiotherapy for at least three months prior to surgery. All surgeries were performed at the Eye Hospital of Wenzhou Medical University, Wenzhou, China. Patient details and histories of antithyroid medication use are provided in Table S3 (Supporting Information). Orbital fibroblasts (OFs) were isolated by mincing tissue pieces and plating them in culture dishes, allowing time for adhesion before immersing in high‐glucose DMEM with 20% FBS and 1% penicillin/streptomycin (P/S). Experiments were conducted with strains between passages 3 and 7, repeated with at least three independent strains.

### Cell Culture

All the cell lines and cell culture reagents are listed and summarized in Table S4 (Supporting Information). All cell lines were cultured at 37°C in a humid atmosphere with 5% CO_2_.

### Adipogenesis

When the OFs were confluent to 100%, the complete growth medium was converted to a differential medium (DM). The base medium for DM was high‐glucose DMEM containing 10% FBS and 1% P/S, supplemented with 0.1 mM indomethacin (Sigma), 0.1 µm dexamethasone (Sigma), 0.5 mm 3‐isobutyl‐1‐methylxanthine (IBMX) (MedChemExpress), and 10 µg mL^−1^ insulin (Procell). The medium was refreshed every 4 to 5 days, and the induction period lasted for 10–14 days, as previously described.^[^ ^58^ ^]^

### Expression Vector Construction and Transfection

Human TSHR cDNA (GeneCopoeia, NM_000369.4) was cloned into a pLVX‐Puro vector (oe‐TSHR) and verified by DNA sequencing. The wild‐type TSHR plasmid was co‐transfected with lentiviral packaging vectors to produce recombinant lentiviral particles, which were used to infect HEK293T cells, followed by puromycin selection to establish TSHR‐overexpressing HEK293T cells. An empty plasmid was similarly packaged into lentiviral particles to generate MOCK cells. Mutant TSHR plasmids were constructed by RIBOBIO CO., Ltd. and transfected into HEK293T cells using Lipofectamine™ 3000 (Invitrogen, USA).

### DNA Library, SELEX Primers, and Buffers

The ssDNA Library used by Cell‐SELEX contained a 42‐nucleotide central random region flanked by two 19‐nucleotide primer‐binding regions. A FAM‐labeled primer (5'‐FAM‐ACCGACCGTGCTGGACTCA‐3') and a biotin‐labeled primer (5'‐biotin‐CGCCAGGCTCGCTCATAGT‐3') were utilized for PCR amplification. The washing buffer was composed of DPBS containing 5 mM MgCl_2_ and 4.5 g L^−1^ of glucose. The binding buffer was formed from the washing buffer containing 0.1 mg/mL yeast tRNA and 1 mg/mL BSA. All DNA sequences listed in Table S1 (Supporting Information) were HPLC‐purified and synthesized in Sangon Biotech (Shanghai, China).

### Cell‐SELEX Procedure

Cell‐SELEX procedures followed published protocols.^[^ ^59^ ^]^ Initially, 10 OD of the ssDNA library was dissolved in binding buffer, denatured at 95 °C for 10 min, and cooled on ice. In the first round, ssDNA was incubated with TSHR‐293T cells at 4 °C for 90 min, washed three times, and the bound ssDNA was released by heating at 95 °C for 10 min. The harvested ssDNA was amplified via PCR using FAM‐labeled forward and biotin‐labeled reverse primers, captured using streptavidin‐coated sepharose beads (GE Healthcare), and isolated after denaturation. From the third round, negative selection with MOCK cells was introduced to enhance specificity. The ssDNA pool was incubated with MOCK cells for 30 min, and unbound ssDNA was collected for positive selection. As the screening rounds progressed, the criteria became increasingly stringent to enhance the affinity and specificity of the sequences. After enrichment, high‐throughput sequencing was performed using Illumina MiSeq by Sangon Biotech (Shanghai, China).

### Flow Cytometric Analysis

Flow cytometry analysis was used to assess the binding ability of evolved ssDNA pools, aptamers, or antibodies to cells. TSHR‐293T and MOCK cells were incubated with FAM‐labeled ssDNA pools at 4 °C for 30 min, washed, and resuspended in the washing buffer for flow analysis. The *K_d_* of the aptamer was calculated using the following equation: Y = *B_max_* X/(*K_d_* +X) by GraphPad Prism 8.0. TSHR expression on various cell membranes was evaluated by incubating cells with FITC‐labeled IgG2a, followed by an anti‐TSHR antibody, and FITC‐conjugated secondary antibodies. Cell fluorescence signals were recorded using a FACS Calibur flow cytometer (BD Bioscience) and analyzed with FlowJo 10.0. All experiments were repeated three times.

### Immunofluorescence Analysis Assay

Immunofluorescence analysis mainly includes the binding ability of aptamers or antibodies to cells or tissues. For aptamers, cells were incubated with fluorescence‐labeled aptamer in 500 µL of binding buffer at 4 °C for 30 min. For antibodies, cells were fixed with 4% paraformaldehyde, washed, and blocked with 10% normal goat serum. Cells were then incubated with primary antibodies overnight at 4 °C. After washing, secondary fluorescent antibodies were incubated, followed by DAPI nuclear counterstaining. Images were acquired using a Cell Observer SD system (Zeiss, Germany).

Paraffin‐embedded tissue sections were prepared, heated at 60 °C, deparaffinized in xylene, and rehydrated through decreasing ethanol concentrations. Epitope retrieval was performed using citrate buffer (pH 6.0). Sections were blocked with DNA blocking buffer (20% FBS, 0.1 mg mL^−1^ salmon sperm DNA), then incubated with anti‐TSHR antibody (1:500, Abcam, ab27974) overnight at 4 °C. After washing, sections were stained with fluorescence‐conjugated secondary antibodies. Sections were then incubated with Cy3‐labeled aptamer Library or YC3, counterstained with DAPI, sealed, and imaged using a fluorescence microscope (Leica, Bannockburn, IL, USA).

### Surface Plasmon Resonance Experiments

Surface Plasmon Resonance (SPR) was used to determine the binding affinity between TSHR and the aptamer YC3 using a BIAcore 8K instrument with CM5 chips at 25 °C. Anti‐His antibodies (Proteintech, 66005‐1‐lg) were immobilized on the CM5 chip via amine coupling to create an anti‐His CM5 chip. TSHR‐His recombinant proteins (Sangon Biotech, China) were then captured by this chip. YC3 was serially diluted to concentrations ranging from 0 to 200 nm and injected at 30 µL min^−1^ for 180 s, followed by 180 s of dissociation. All experiments were conducted in running buffer (DPBS with 5 mM Mg^2^⁺, pH 7.4). The relative response was recorded and fitted to a 1:1 kinetic model using Biacore evaluation software.

### Structures of Protein and Aptamer

The protein structures of TSHR and M22 were obtained from the RCSB Protein Data Bank (PDB IDs: 7XW5, 7XW6).^[^ ^10^ ^]^ The Vienna number of aptamer YC3 was generated online using the MFOLD web server,^[^ ^60^ ^]^ and the corresponding 3D structure with the best‐predicted energy was constructed using 3dRNA/DNA.^[^ ^61^ ^]^ To eliminate steric conflicts within the aptamer system, a position‐constrained molecular dynamics simulation was performed.

### Molecular Docking

Aptamer YC3 and protein TSHR were subjected to molecular docking using Rosetta software,^[^ ^62^ ^]^ involving two steps: aggressive sampling with a centroid model followed by fine optimization using a full‐atom model. Initially, rigid‐body blind docking generated at least 2,000 conformations with position‐restrained heavy atoms and randomly oriented protein and aptamer. Amino acids forming frequent hydrogen bonds (>0.4 frequency) were selected as potential binding sites, excluding distant sites. In the fine optimization step, the aptamer was positioned within 10 Å of the binding pockets and perturbed by a 3 Å translation and an 8° rotation before each simulation. Side chains of protein residues and the complete DNA were allowed to move. Up to 100 conformers were assessed, and the one with the lowest binding energy was selected for subsequent molecular dynamics simulation.

### Molecular Dynamics Simulations

The optimal docking conformation of the TSHR‐YC3 complex from Rosetta docking served as the starting point for molecular dynamics (MD) simulations using Amber 20 software.^[^ ^63^ ^]^ Simulations lasted at least 100 ns, with RMSD monitored until system equilibrium was achieved. The protein system utilized the GAFF and ff14SB force fields, enclosed in a cubic water box with a 10 Å radius around the protein, and Na^+^ ions were added to neutralize the system. The simulation process included: (1) Two‐step energy minimization; (2) System equilibration; and (3) Dynamics simulation. The cutoff distance for van der Waals and short‐range electrostatic interactions was set to 10 Å, and the Particle‐Mesh‐Ewald method was used to calculate long‐range electrostatic interactions. In this system, the average structure from the time interval of 60–80 ns during the equilibrium state was extracted for subsequent binding mode analysis.

### Circular Dichroism Experiments

Circular dichroism experiments to assess structural changes in TSHR‐LRD protein were conducted using a Chirascan‐plus spectropolarimeter (Applied Photophysics, UK). The protein (0.222 µM) was diluted in 1× DPBS with 5 mM MgCl_2_, which also served as the blank. Measurements were taken at 298 K using a 0.5 mm quartz cuvette, scanning from 200–300 nm (1 nm bandwidth, 100 nm min^−1^ speed), with each spectrum averaged over three scans. To eliminate contributions of YC3 in a CD spectrum, the same concentration of YC3 (without TSHR‐LRD) was subtracted from the respective CD spectra.

### Establishment of the GO Mouse Model by Immunization with Ad‐TSHR289

Female BALB/c mice were purchased from Weitong Lihua Experimental Animal Technology Co., Ltd. and housed in a pathogen‐free environment at the Eye Hospital of Wenzhou Medical University. Mice were randomly divided into three groups: GO, MOCK, and Blank. The GO group received intramuscular injections of 2 × 10^9^ particles of Ad‐TSHR289 in 50 µL PBS, the MOCK group received an equal number of particles of empty‐vector adenovirus in 50 µL PBS, and the Blank group received 50 µL PBS. Injections were administered every three weeks for a total of nine injections, with all mice sacrificed at week 25. The mice were maintained in a controlled environment with a 12‐h light/dark cycle, had unrestricted access to water and food, and were weighed every three weeks.

### Administration Regimen in GO Mice

After constructing the GO models for 18 weeks, T3, T4, and TRAb serum levels in the GO group were measured, with successful model construction defined as levels more than twice those of the Blank group. Successfully modeled GO mice were randomly divided into four groups. From weeks 18 to 24, mice were anesthetized with isoflurane and received periorbital injections three times per week. Each eye was injected with either PBS, aptamer PS‐YC3‐PEG (10 mg kg^−1^), aptamer PS‐Library‐PEG (10 mg kg^−1^), or DSP at 1.5 mg kg^−1^. The treatment regimen is illustrated in Figure 7A. The appearance of the mice's eyes was observed and photographed, and their body weight was recorded weekly during the treatment period.

### In Vivo Imaging

Aptamers Cy5‐labeled PS‐Library‐PEG and Cy5‐labeled PS‐YC3‐PEG were administered to GO mice via periorbital injection at a dose of 2.24 mg kg^−1^. The distribution of the aptamers was monitored within 48 h using an IVIS Lumina II imaging system, with excitation and emission wavelengths set at 640 and 670 nm, respectively. Two hours post‐injection, the mice were euthanized, organs were dissected, and fluorescence accumulation in the tissues was imaged.

### Pharmacokinetic Studies

Pharmacokinetic studies were conducted in female BALB/c mice (8 weeks old, 20 ± 2 g). Cy5‐labeled PS‐YC3‐PEG was administered via periorbital injection at a dose of 1.12 mg kg^−1^. Blood samples were collected from the contralateral retro‐orbital venous plexus at 5, 15, and 30 min, and at 1, 2, 4, 8, and 12 h post‐injection under isoflurane anesthesia. After allowing the blood to stand at room temperature for 15 min, plasma was obtained by centrifugation at 5000 rpm for 10 min. A 10 µL aliquot of plasma was diluted six‐fold and transferred to a black‐walled, clear‐bottom 96‐well plate. Fluorescence intensity was then measured using an IVIS Lumina II imaging system with excitation and emission wavelengths set at 640 nm and 670 nm, respectively.

### Statistical Analysis

Data are presented as mean ± SEM from at least three independent experiments per group, with the exact number indicated in the figures and legends. Statistical analyses of experimental results were conducted using GraphPad Prism v10. Two‐tailed unpaired Student's t‐tests were used for comparisons between two groups, while one‐way analysis of variance (ANOVA), followed by Tukey's multiple post hoc test, was employed to assess differences among multiple groups. A two‐way ANOVA was employed to evaluate the effects of two independent variables on a continuous outcome measure. A P value less than 0.05 was considered statistically significant.

## Conflict of Interest

The authors declare no conflict of interest.

## Author Contributions

Y.Z., E.W., and W.L. contributed equally to this work. W.W. and M.Y. designed the research; Y.Z., E.W., and W.L. analyzed the data; Y.Z., E.W., W.L., and Z.L. performed the experiments and collected the data; Y.Z., E.W., N.L., L.Z., H.W., S.Y., T.P., Y.H., X.L., W.Y., and Y.T. performed the investigation; Y.Z. drafted the original manuscript; J.W., M.Y., and W.W. conceived and supervised the study and revised the manuscript. All authors read and approved the final manuscript.

## Supporting information



Supporting Information

## Data Availability

The data that support the findings of this study are available from the corresponding author upon reasonable request.
