# Supplementary material for: TSHR‐Targeting Nucleic Acid Aptamer Treats Graves' Ophthalmopathy via Novel Allosteric Inhibition
Source: Adv Sci (Weinh). 2025 Oct 7;13(4):e05586. doi: 10.1002/advs.202505586 (PMC12822413; doi:10.1002/advs.202505586)
Supplement: Supplementary file 1 — Supporting Information [file ADVS-13-e05586-s001.docx]

Title: TSHR-targeting Nucleic Acid Aptamer Treats Graves' Ophthalmopathy via Novel Allosteric Inhibition

**Authors:** Yanchen Zhang^1,3,4^†, Ende Wu^1,3^†, Weibin Liu^1,4^†, Ling Zeng^1,4^, Neng Ling^1,3,4^, Hongmei Wang^1^, Zhixing Li^1^, Shuang Yao^1^, Tonghe Pan^1^, Xuanwen Li^1^, Yate Huang^1^, Xiaojing Li^1^, Yunhai Tu^1^, Wentao Yan^1^, Jianzhang Wu^3,1,4^*, Mao Ye^2^*, and Wencan Wu^3,1,4^*

**Supplementary Materials and Methods**

*Aptamer-mediated Pull-down Assay:* The pull-down assay was conducted as previously described.^[1]^ 3×10^7^ TSHR-293T cells were dissociated with 0.2% EDTA, collected by centrifugation, and membrane proteins were extracted using a kit (PH0710, Phygene). The membrane proteins were divided into four parts. Three of these parts were incubated in a DNA-blocking buffer at 4°C. Two of these parts were then incubated with biotin-labeled aptamer Library (control) or biotin-labeled aptamer YC3 overnight at 4°C. Then these three parts were incubated with streptavidin-coated magnetic beads at 4°C. After centrifugation and washing, the samples were treated with protein loading buffer, heated, and subjected to SDS-PAGE electrophoresis followed by Western blotting.

*Small Interfering RNA Interference and Transfection:* Small interfering RNA (siRNA) interference was conducted according to the published protocol.^[2]^ siRNA sequences targeting TSHR and a random sequence (listed in Table S5) were synthesized by RIBOBIO CO., Ltd. (Guangzhou, China). In brief, TSHR-293T cells were transfected with siRNA by Lipofectamine™ 3000 transfection reagent (Invitrogen, USA).

*PEGylation of Aptamer:* PEGylation was performed according to previously described methods.^[3]^ Specifically, the thiol-labeled aptamer was suspended in PBS and reduced with 20 mM DTT (PHYGENE, China). After the reaction, the DTT was removed using spin filters. The reduced aptamers were then incubated with an excess amount of 10 kDa maleimide-terminated polyethylene glycol (Mal-PEG) (Ponsure Biotechnology, Shanghai, China). Following conjugation, unconjugated Mal-PEG was removed using spin filters.

*Measurement of cAMP Production:* Cells were seeded in 24-well plates and starved with 1% FBS overnight. The next day, cells were incubated in antibiotic-free serum-free media containing 0.5 mM IBMX to avoid cAMP decay, followed by adding aptamers, with or without M22 (100 ng/mL). The intracellular extracts were gathered and stored at -80°C until the cAMP analysis was conducted using the cAMP assay (KGE002B, R&D Systems, USA). The assay employs polyclonal antibodies to competitively bind cAMP in both standard and sample extracts, as described previously.^[4]^

*RNA Isolation, Reverse Transcription and Quantitative PCR:* Total RNA from cells and adipose tissues was extracted using TRIzol reagent (Invitrogen) following the manufacturer's instructions. RNA concentration was measured with a UV spectrophotometer. cDNA was synthesized using a reverse transcription kit (Promega, USA), and real-time PCR was conducted with PCR SYBR Green (APExBIO, USA). Three independent experiments were performed to ensure reliability. Primer pairs were synthesized by Tsingke Biotech and detailed in Supplementary Table S6.

*Western Blotting:* Total protein was extracted using RIPA buffer (EpiZyme) with protease inhibitor cocktail, and concentrations were determined using a protein assay reagent (EpiZyme). Protein samples were separated on SDS-PAGE gels and transferred to nitrocellulose membranes using a Bio-Rad Western blot transfer system. Membranes were washed with TBST, blocked with 5% skim milk and incubated with primary antibodies overnight at 4°C. After washing, membranes were incubated with Goat anti-Mouse IgG (1:5000, LI-COR, IRDye680CW). Detection was performed using an Odyssey infrared scanning system (LI-COR). Primary antibodies used included: anti-TSHR (Abcam), anti-β-actin (Proteintech), anti-GAPDH (Bioss), anti-ERK1/2 (Cell Signaling Technology), anti-phospho-ERK1/2 (Cell Signaling Technology), anti-MEK1/2 (Cell Signaling Technology), anti-phospho-MEK1/2 (Cell Signaling Technology) and anti-PPARγ (Proteintech). All primary antibodies were used at a dilution of 1:1000.

*Enzyme-linked Immunosorbent Assay:* The concentrations of inflammatory cytokines (IL-6, IL-8, and IL-1β) in cell-conditioned supernatants were measured using ELISA kits (FineTest, Wuhan, China): EH0201 for IL-6, EH0205 for IL-8, and EH0185 for IL-1β, following the manufacturer's protocols. Similarly, T3, T4, and TRAb concentrations in mouse serum from the orbital venous sinus were measured using ELISA kits (FineTest): EU0403 for T3, EU0402 for T4, and EM1423 for TRAb. The concentration of HA was quantified using a specific ELISA kit from Echelon Biosciences (Salt Lake City, UT), according to the manufacturer’s instructions.

*Histopathological Examination:* For H&E staining, sections were stained with hematoxylin, rinsed, differentiated, and rinsed again. Slides were then immersed in ammonia solution, rinsed, dehydrated with 85% and 95% ethanol, and stained with eosin. After dehydration with a gradient of alcohol, slides were cleared with xylene and sealed with neutral gum. For Masson staining, follow the instructions provided with the Masson staining kit (Beyotime, China). For periodic acid-schiff (PAS) staining, follow the instructions provided with the PAS staining kit (Beyotime, China). The image data were collected using a Pannoramic Slide Scanner (3DHISTECH, Budapest, Hungary) and DM4B biological microscope (Leica, Bannockburn, IL, USA). Adipose tissue around the optic nerve and the average area of adipocytes were quantified using Fiji/ImageJ software. The orbital fat area was normalized to the optic nerve area in each mouse. The area fraction of collagen or glycogen was calculated using Fiji/ImageJ software as follows: Area fraction of collagen or glycogen (%) = (average collagen or glycogen area/total field area) × 100.

*IHC Analysis of Tissues:* Tissue sections were deparaffinized with xylene and rehydrated through graded ethanol solutions. Antigen retrieval was performed in citrate buffer (pH 6.0), followed by blocking endogenous peroxidase at room temperature. Sections were blocked with 10% normal goat serum for 1 hour, then incubated with primary antibody overnight at 4°C, and secondary HRP-conjugated antibodies for 1 hour at room temperature. Staining was achieved using 3,3'-Diaminobenzidine Tetrahydrochloride (DAB), followed by hematoxylin counterstaining for 3 minutes. Sections were dehydrated with alcohol and cleared with xylene before being sealed with neutral gum. Slides were imaged using a DM4B biological microscope (Leica, Bannockburn, IL, USA) and the integrated optical density (IOD) was measured using Fiji/ImageJ software.

*Oil Red O Staining:* Oil Red O staining was performed using the ORO kit (PHYGENE, China) according to the manufacturer's instructions. Staining was inspected and photographed using an inverted microscope (Nikon, Japan). For quantification, the staining was eluted from the cells with isopropanol, and the optical density (OD) was measured at 490 nm using a microplate reader (Molecular Devices), as described before.^[5]^

*Magnetic Resonance Imaging:* MRI was performed using a 9.4 Tesla animal scanner (BioSpec 94/20 USR, Bruker BioSpin, Ettlingen, Germany) with ParaVision 3.2 software. A 3-channel phased-array surface coil (Bruker) served as the receiver, and an 86-mm diameter volume coil (Bruker) was used as the transmitter. T2-weighted images were acquired using a Turbo RARE sequence with the following parameters: Field of View=2.0 × 2.0 cm², Matrix Size=256 × 256, Slice Thickness=0.30 mm, Interslice Distance=0.0 mm, Number of Slices=20, TR/TE=2500/33 ms. Region of interest (ROI) analysis of MRI images was performed using the Medical Imaging Interaction Toolkit (MITK) software.^[6]^ Briefly, the workflow included importing DICOM image data into MITK, applying N4 bias correction and denoising for image enhancement, and segmenting key orbital structures, including globes, nerves, extraocular muscles, and adipose tissue, using manual, semi-automated, or atlas-based segmentation techniques. Quantitative analysis was performed for area measurements, volumetric measurements and signal intensity analysis.

**Table S1. Sequences of synthesized oligonucleotides used in the experiments**

| Sample | Sequence (5’→3’) |
| --- | --- |
| Library | ACCGACCGTGCTGGACTCANNNNNNNNNNNNNNNNNNNNNNNNNNNNNNNNNNNNNNNNNNACTATGAGCGAGCCTGGCG |
| YC1 | ACCGACCGTGCTGGACTCAGGGCGCATCTTTGGTAAGCAAAGATGGGTTGCAAGCCTCTCGACTATGAGCGAGCCTGGCG |
| YC2 | ACCGACCGTGCTGGACTCAATCCATCGTCGACGAGCATACGAAGGGAGGGTATTGTAAGGGACTATGAGCGAGCCTGGCG |
| YC3 | ACCGACCGTGCTGGACTCAGATGAACAATCGATGGAAGATTGGGTCGCACCGGCCTCTTTCACTATGAGCGAGCCTGGCG |
| YC4 | ACCGACCGTGCTGGACTCAGGGTGGGTCGTTTCATAGGAAACGGGTGGAACGCCTCTAGCCACTATGAGCGAGCCTGGCG |
| YC5 | ACCGACCGTGCTGGACTCAGACGGAATCGTCCTTTGTCTACGTGCGGTCTAGACTGTATAGACTATGAGCGAGCCTGGCG |
| YC6 | ACCGACCGTGCTGGACTCAGCCGTGACGTTCTACAGAGAACGGGTGGATCCCGCCTCTTCACTATGAGCGAGCCTGGCG |
| YC7 | ACCGACCGTGCTGGACTCATGGTCACATCTATCGTACTAGCATGGGTCAATTGGCCTCTTGACTATGAGCGAGCCTGGCG |
| YC8 | ACCGACCGTGCTGGACTCAGCCGTGACGTTCTACAGAGAACGGGTGGACCCGCCTCCTCACTATGAGCGAGCCTGGCG |
| YC3-a | ACCGACCGTGCGATGAACAATCGATGGAAGATTGGGTCGCACCGGCCTCTTTCACTATGAGCGAGCCTGGCG |
| YC3-b | ACCGACCGTGCTGGACTCAGATGAACTGGGTCGCACCGGCCTCTTTCACTATGAGCGAGCCTGGCG |

**Table S2. Binding free energy decomposition between TSHR and aptamer YC3**

| **Residues** | **Binding free energy (kcal/mol)** |
| --- | --- |
| K146 | -2.67 |
| N170 | -3.18 |
| Q173 | -1.39 |
| T190 | -2.46 |
| Q193 | -2.47 |
| Y195 | -2.67 |
| N198 | -1.87 |
| K218 | -3.51 |
| D219 | -3.46 |
| S243 | -1.06 |
| K244 | -3.23 |
| L265 | -1.23 |
| L266 | -1.36 |
| S268 | -2.94 |

**Table S3. Demographic information of the donors recruited in the study**

| **Age, year/Sex** | **Duration of GO [year]** | **Smoking Status** | **Previous Steroid Use** | **Radiation** | | **Thyroid Treatment** |
| --- | --- | --- | --- | --- | --- | --- |
| 33/M | 1 | No | Oral Prednisone | None | No | |
| 57/F | 10 | No | Oral Prednisone | None | Methimazole | |
| 59/M | 1 | Previous | None | None | Thyroidectomy | |
| 50/M | 0.25 | Previous | None | None | Methimazole | |
| 36/M | 0.75 | Previous | None | None | Thyroidectomy | |
| 55/F | 3 | No | Oral Prednisone | None | Thyroidectomy | |
| 20/F | N/A | No | N/A | N/A | N/A | |
| 22/F | N/A | No | N/A | N/A | N/A | |
| 25/F | N/A | No | N/A | N/A | N/A | |
| 24/F | N/A | No | N/A | N/A | N/A | |
| 21/F | N/A | No | N/A | N/A | N/A | |
| 24/M | N/A | Previous | N/A | N/A | N/A | |

F, female; M, male; GO, Graves’ ophthalmology; CAS, clinical activity score; N/A, not applicable.

**Table S4. Culture conditions and sources of cell lines**

| Cell | Culture condition | Cell Sources |
| --- | --- | --- |
| Human embryonic kidney 293T cells (HEK293T) | high-glucose DMEM (Gibco) containing 10% FBS and 1% penicillin-streptomycin | Procell Life Science & Technology (Wuhan, China) |
| Human follicular epithelial cells (Nthy-ori 3-1) | RPMI-1640 medium (Gibco) containing 10% FBS and 1% penicillin-streptomycin | Procell Life Science & Technology (Wuhan, China) |
| Human thyroid cancer cells (FTC-133) | RPMI-1640 medium (Gibco) containing 10% FBS and 1% penicillin-streptomycin | Wenzhou Medical University |
| Human thyroid cancer cells (BCPAP) | RPMI-1640 medium (Gibco) containing 10% FBS and 1% penicillin-streptomycin | Wenzhou Medical University |
| Human corneal epithelial cells (HCECs) | high-glucose DMEM (Gibco) containing 10%FBS and 1% penicillin-streptomycin | Wenzhou Medical University |
| Human lens epithelial cells (HLECs) | high-glucose DMEM (Gibco) containing 10% FBS and 1% penicillin-streptomycin | Wenzhou Medical University |
| Human retinal endothelial cells (HRECs) | high-glucose DMEM (Gibco) containing 10% FBS and 1% penicillin-streptomycin | Wenzhou Medical University |
| Adult retinal pigment epithelial cell line-19 (ARPE19) | Dulbecco’s Modified Eagle Medium (DMEM), consisting of nutrient Mixture F-12 media (Gibco) containing 10% FBS and 1% penicillin-streptomycin | Wenzhou Medical University |
| The human microglial clone 3 cells (HMC3) | minimum essential medium (Gibco) containing 10% FBS and 1% penicillin-streptomycin | Wenzhou Medical University |

**Table S5. siRNA and random sequences**

| Name | Sequence (5’→3’) |
| --- | --- |
| TSHR siRNA-1 sequences | GTACAACAATGGCTTTACT |
| TSHR siRNA-2 sequences | TGACGTCAATCCCTGTGAA |
| Negative control | UUCUCCGAACGUGUCACGUTT |

**Table S6. Primers used for qPCR**

| Name | Sequence (5’→3’) |
| --- | --- |
| TSHR | F: CCATCAGGAGGAGGACTTCA  R: ATTGGGCAGATTAGAAAATG |
| GAPDH | F: AAATCAAGTGGGGCGATGCTG  R: GCAGGAGGCATTGCTGATGAT |
| IL-6 | F: GTACATCCTCGACGGCATC  R: ACCTCAAACTCCAAAAGACCAG |
| IL-8 | F: GAGAGT GATTGAGAGTGGACC  R: ACTGATTCTTGGATACCACAGAG |
| IL-1β | F: GGCTTATTACAGTGGCAATG  R: GTAGTGGTGGTCGGAGAT |
| HAS1 | F: TCAAGGCGCTCGGAGATTC  R: CTACCCAGTATCGCAGGCT |
| HAS2 | F: GATGACCTACGAAGCGATT  R: GCCTGCCACACTTATTGA |
| HAS3 | F: CGCAGCAACTTCCATGAGG  R: AGTCGCACACCTGGATGTAGT |
| ACTB | F: CCCTGGAGAAGAGCTACGAG  R: CGTACAGGTCTTTGCGGATG |
| C/EBPα | F: CACCTGCAGTTCCAGATCG  R: CTTGTCCACCGACTTCTTGG |
| PPARγ | F: ATGGCATCCAGAACAAGGAG  R: TCCCGTCTTTGTTCATCACA |

F, forward; R, reverse.


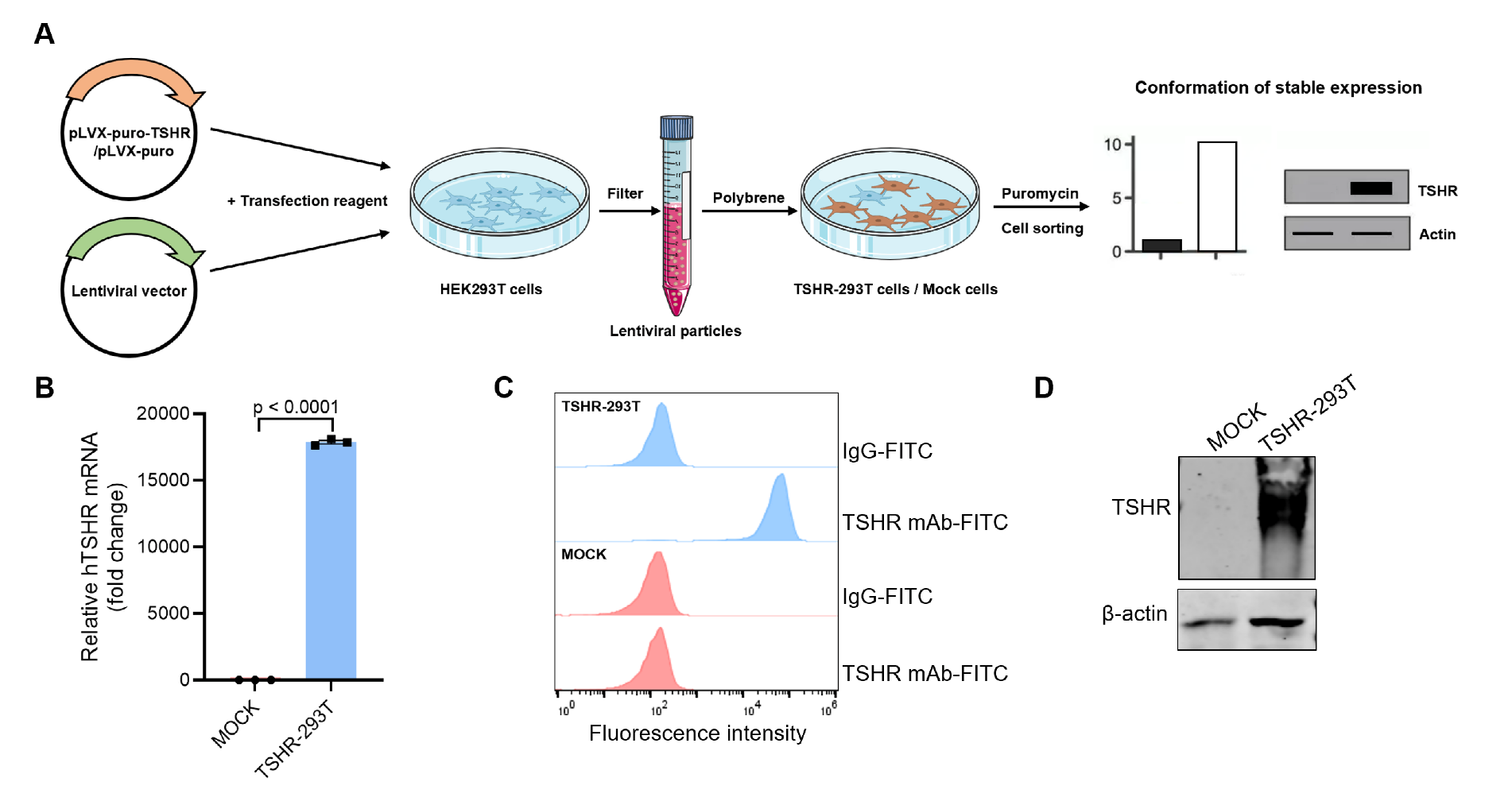


**Figure S1.** Construction and verification of the Cell-SELEX model cells. a) Schematic illustration of the construction of MOCK cells and TSHR-293T cells. b) The relative expression of TSHR mRNA in MOCK cells and TSHR-293T cells was analyzed by qPCR (n = 3). c) Flow cytometry analysis of the expression of TSHR in MOCK cells and TSHR-293T cells. The FITC-labeled IgG was used as the control. d) Representative immunoblot analysis of TSHR protein level in MOCK cells and TSHR-293T cells. β-actin was used as a loading control. All data are represented as the mean ± SEM. Two-tailed unpaired Student’s t-tests were used to calculate P values. Accurate P values are listed in the figures.


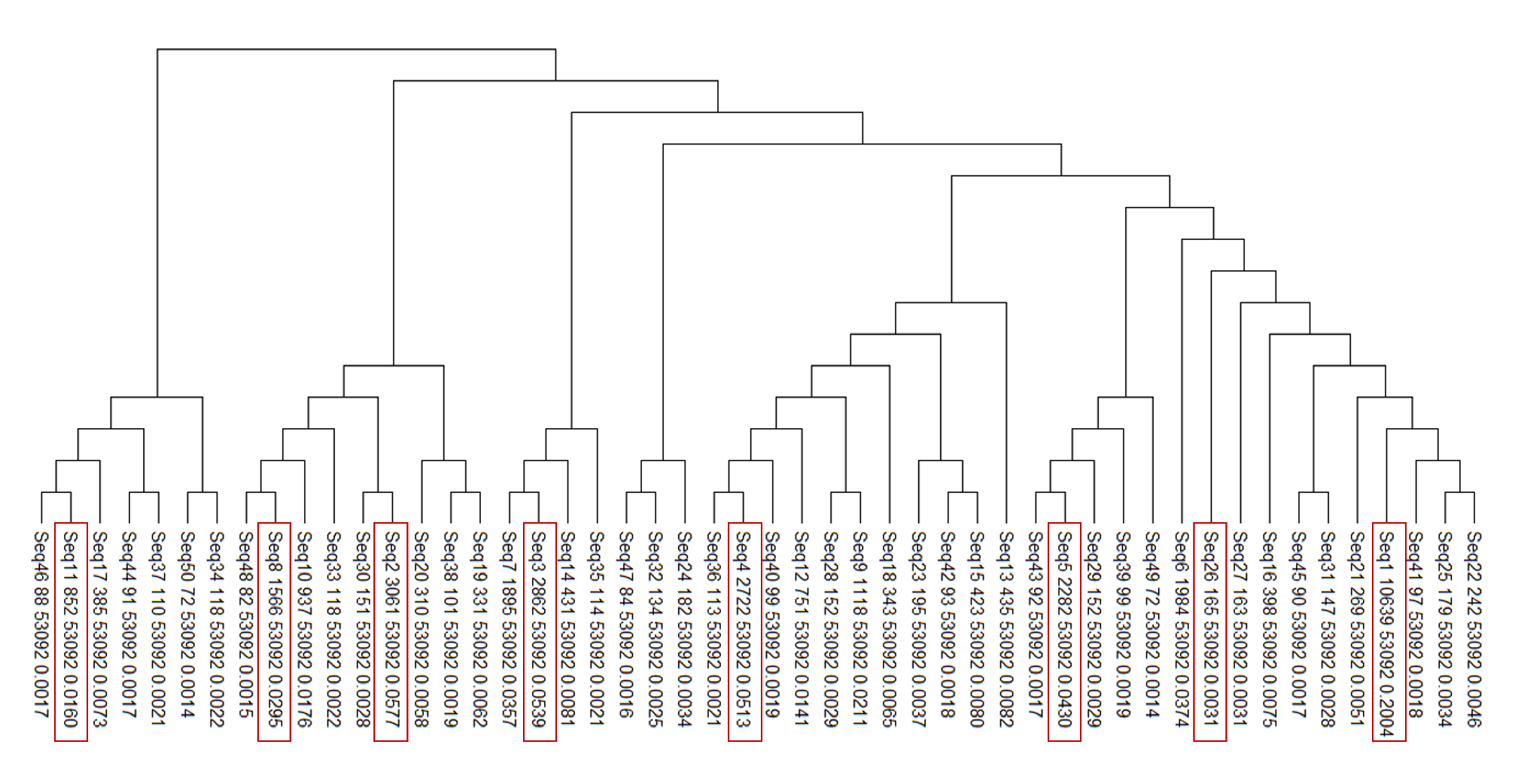


**Figure S2.** Sequence similarity analysis. Dendrogram of similarity visualization classification (sequence name, number of occurrences, total number of occurrences, and frequency of occurrences from top to bottom) for the 50 individual sequences cloned after the 9th round of screening. Eight representative sequences were selected from different families (boxed).


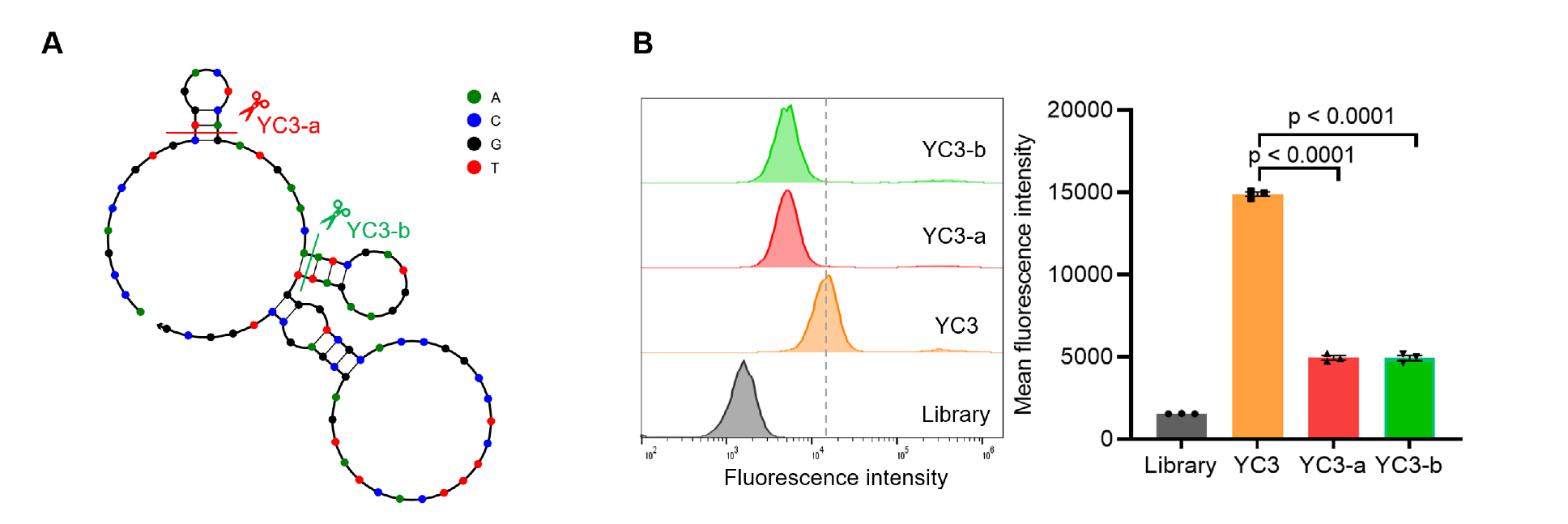


**Figure S3.** Truncation of aptamer YC3. a) Schematic illustration of two truncated versions of YC3. Secondary structures were predicted by the NUPACK software. b) The binding of truncated aptamers (500 nM) to TSHR-293T cells was analyzed by flow cytometry. Quantitative analysis of the relative fluorescence intensity of the aptamers in TSHR-293T cells (n = 3). All data are represented as the mean ± SEM. Two-tailed unpaired Student’s t-tests were used to calculate P values. Accurate P values are listed in the figures.


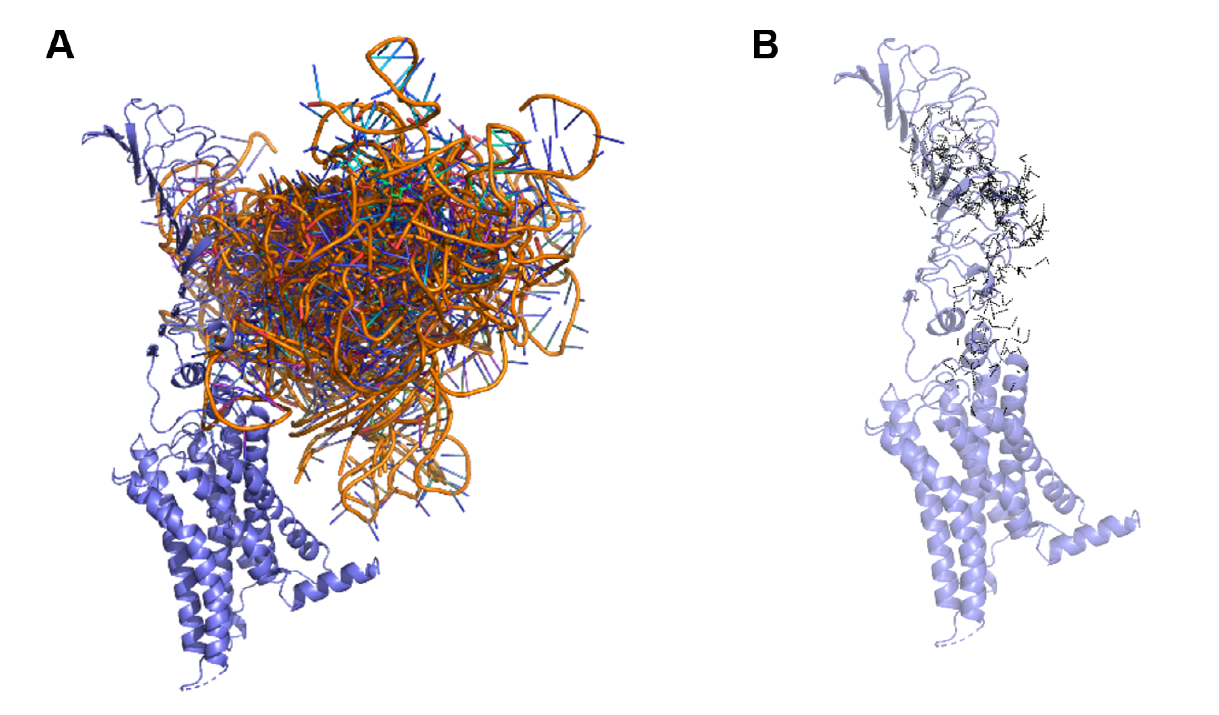


**Figure S4.** Molecular dynamics simulations of TSHR and YC3 docking phases. a) Molecular dynamic simulation results of binding sites between TSHR and the fifty YC3 docking conformations. b) Hydrogen bond network formed between TSHR and all YC3 docking conformations.


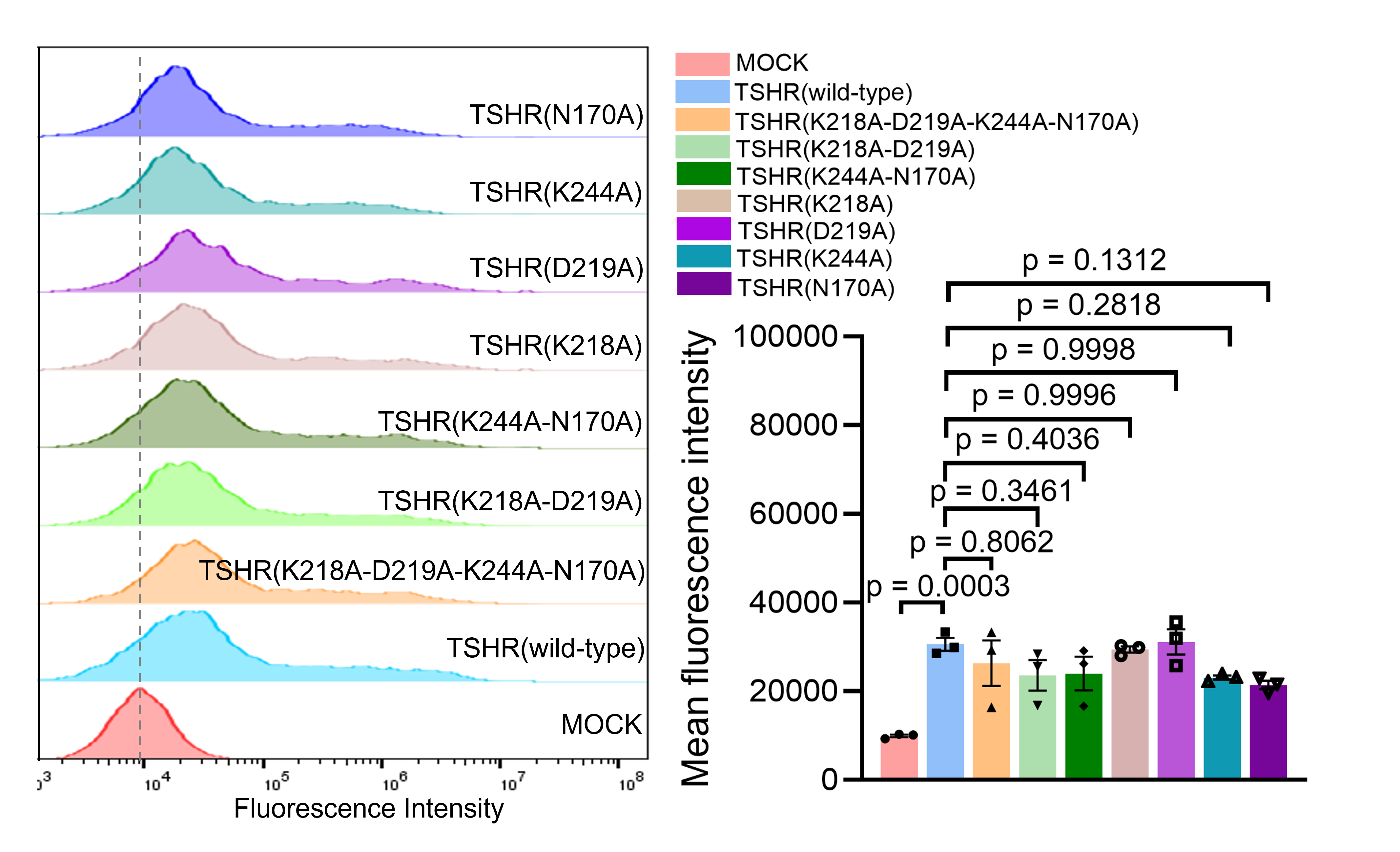


**Figure S5.** Flow cytometry results of TSHR protein expression in HEK293T cells transfected with wild-type and mutant plasmids of TSHR. Quantitative analysis of the relative fluorescence intensity (n = 3). Data are represented as mean ± SEM. One-way ANOVA, followed by Tukey's multiple post hoc test, was used to calculate P values. Accurate P values are listed in the figures.


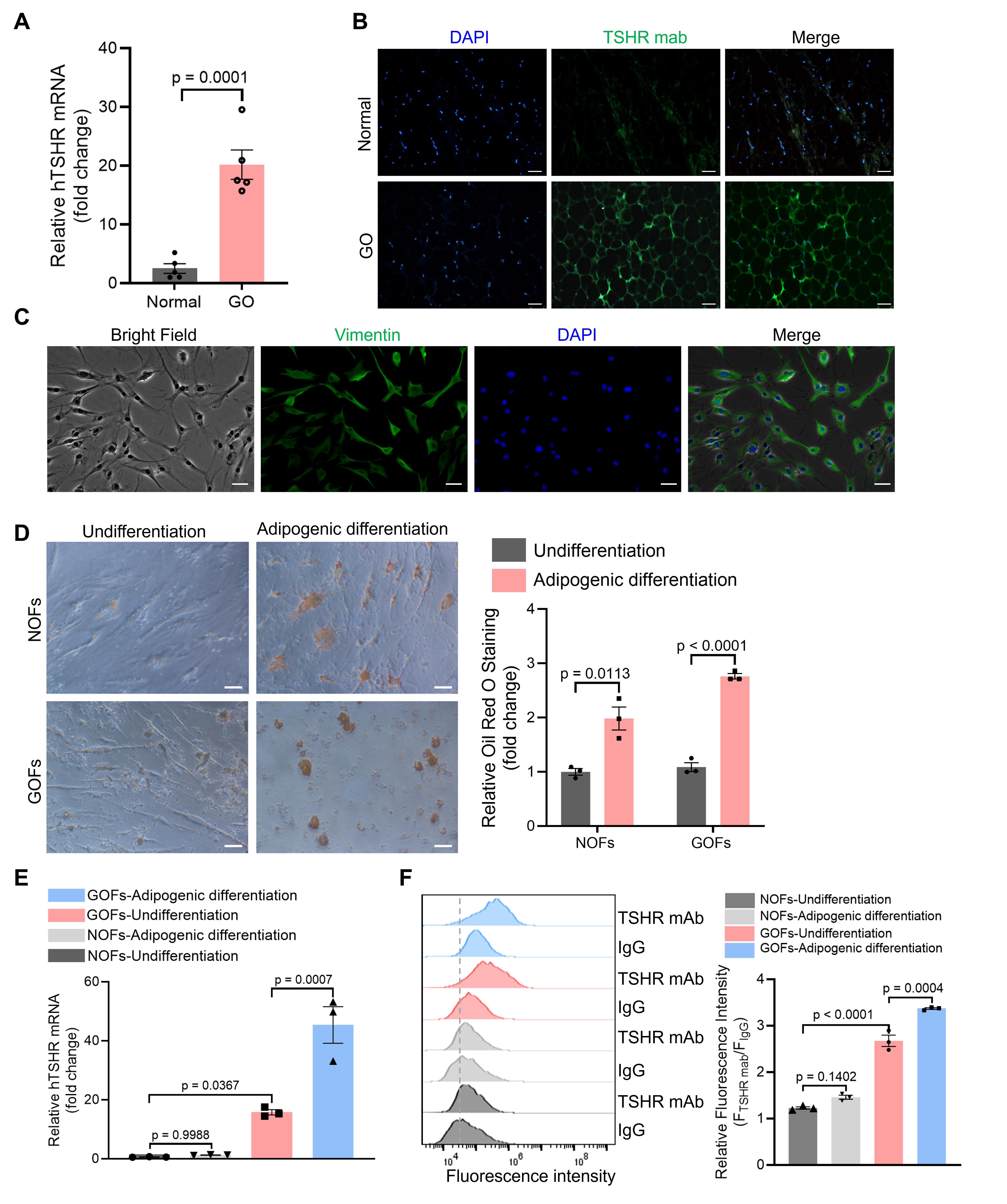


**Figure S6.** TSHR relevance in GO. a) The mRNA expression levels of TSHR in human orbital adipose tissue from healthy donors or patients with GO (n = 5). b) Representative images of immunofluorescence staining on human orbital adipose tissue sections from healthy donors or patients with GO for TSHR (green) and DAPI (blue). Scale bars: 50 μm. c) Representative images of immunofluorescence staining on orbital fibroblasts from human orbital tissue for Vimentin (green), and DAPI (blue). Scale bars: 50 μm. d) Representative images of Oil Red O staining on GOFs and NOFs, both with and without adipogenic differentiation. Scale bars: 50 μm. Quantitative analysis of the relative Oil Red O staining values of cells (n = 3). e) The mRNA expression levels of TSHR in GOFs and NOFs, both with and without adipogenic differentiation (n = 3). f) Flow cytometry analysis of the expression of TSHR in GOFs and NOFs, both with and without adipogenic differentiation. Quantitative analysis of the relative fluorescence intensity (n = 3). All data are represented as mean ± SEM. Two-tailed unpaired Student’s t-tests (A, D) and one-way ANOVA, followed by Tukey's multiple post hoc test (E, F), were used to calculate P values. Accurate P values are listed in the figures.


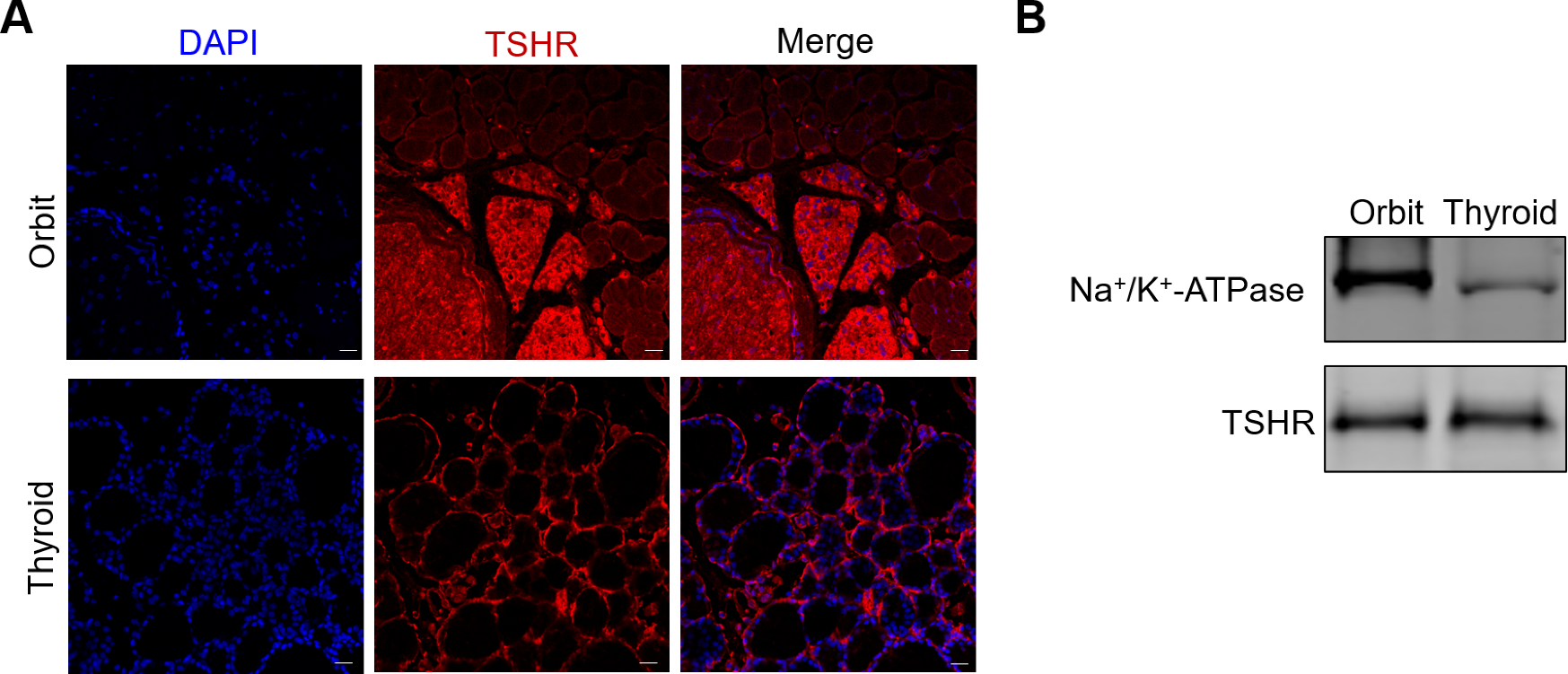


**Figure S7.** Expression of TSHR in mouse thyroidal and orbital tissues. a) Representative immunofluorescence staining images of TSHR (red) and DAPI (blue) in mouse thyroidal and orbital tissue sections. Scale bars: 20 μm. b) Representative immunoblots/densitometric quantitative analysis of TSHR protein levels in mice thyroidal and orbital tissues. Na^+^/K^+^-ATPase was used as a loading control (n = 3). Data are represented as mean ± SEM. Two-tailed unpaired Student’s t-tests were used to calculate P values. Accurate P values are listed in the figures.


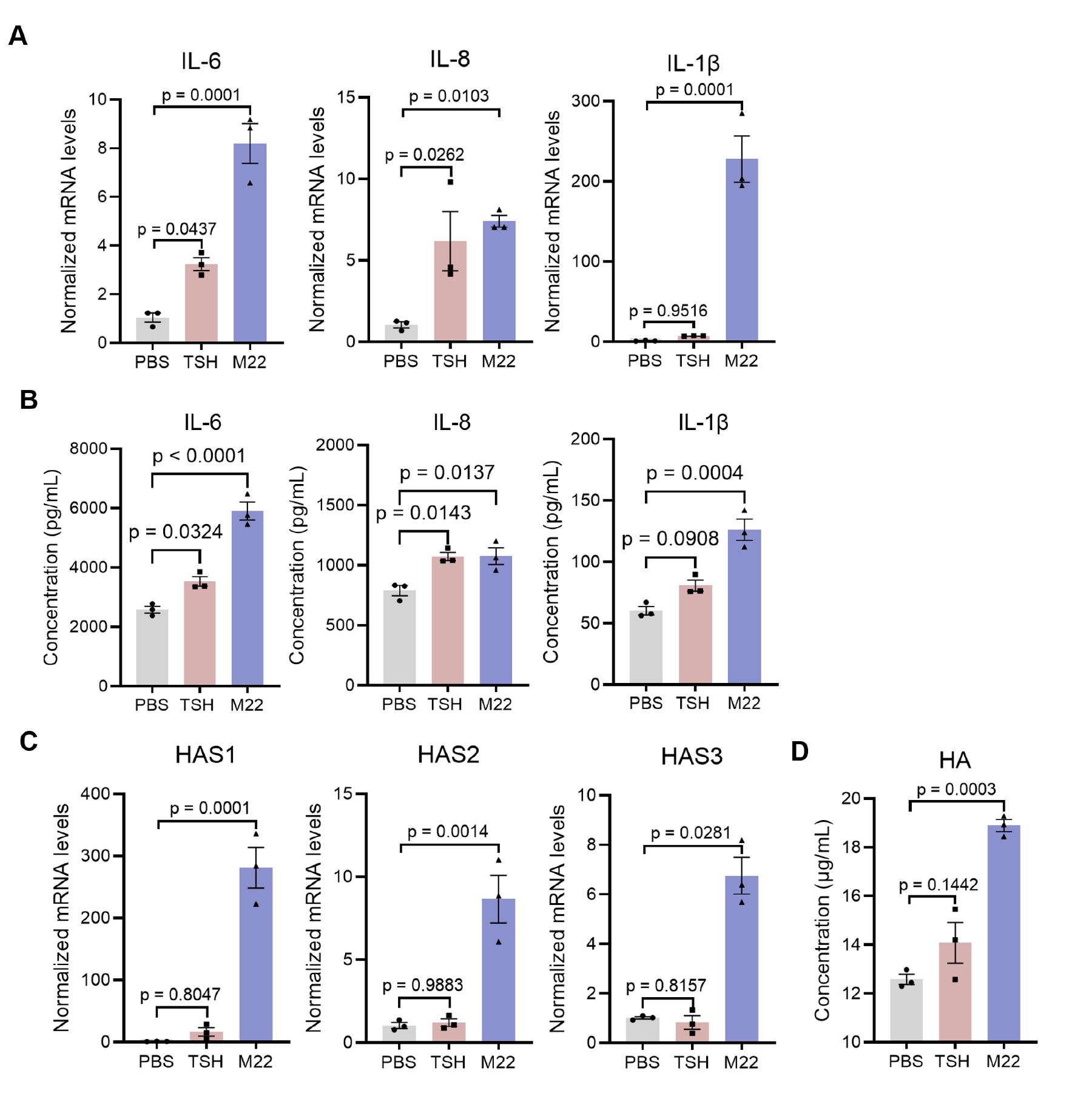


**Figure S8.** Stimulation of TSHR leads to the production of inflammatory cytokines and hyaluronan (HA) in GOFs. a) The mRNA expression levels of IL-6, IL-8, and IL-1β in GOFs stimulated by TSH (100 ng/mL) or M22 (100 ng/mL) by qPCR (n = 3). b) The concentrations of IL-6, IL-8, and IL-1β in the supernatant medium of GOFs stimulated by TSH (100 ng/mL) or M22 (100 ng/mL) were measured by ELISA (n = 3). c) The mRNA expression levels of HAS1, HAS2, and HAS3 in GOFs stimulated by TSH (100 ng/mL) or M22 (100 ng/mL) by qPCR (n = 3). d) The concentrations of HA in the supernatant medium of GOFs stimulated by TSH (100 ng/mL) or M22 (100 ng/mL) were measured by ELISA (n = 3). All data are represented as mean ± SEM. One-way ANOVA, followed by Tukey's multiple post hoc test, was used to calculate P values. Accurate P values are listed in the figures.


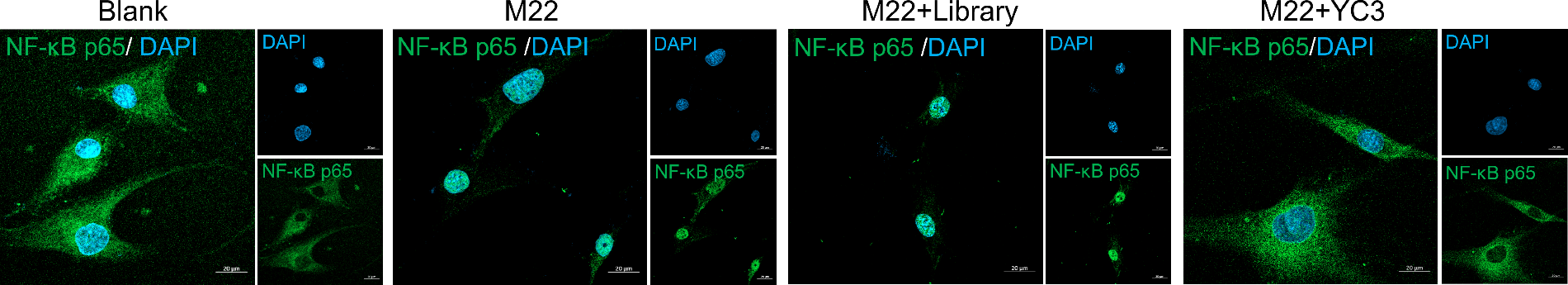


**Figure S9.** Confocal imaging of NF-κB p65 in GOFs stimulated by M22 and treated with either an aptamer Library or YC3 (2 μM). Representative images were shown. Scale bars: 20 μm.


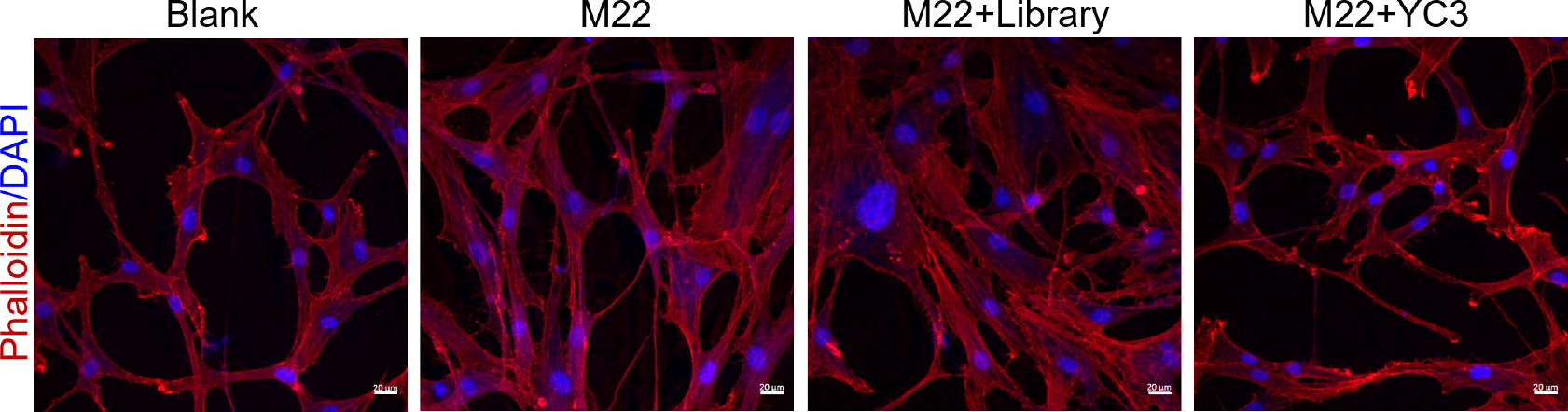


**Figure S10.** Phalloidin staining for actin filaments in GOFs stimulated by M22 and treated with aptamers Library or YC3 (2 μM). The cells were counterstained with DAPI. Representative images were shown. Scale bars: 20 μm.


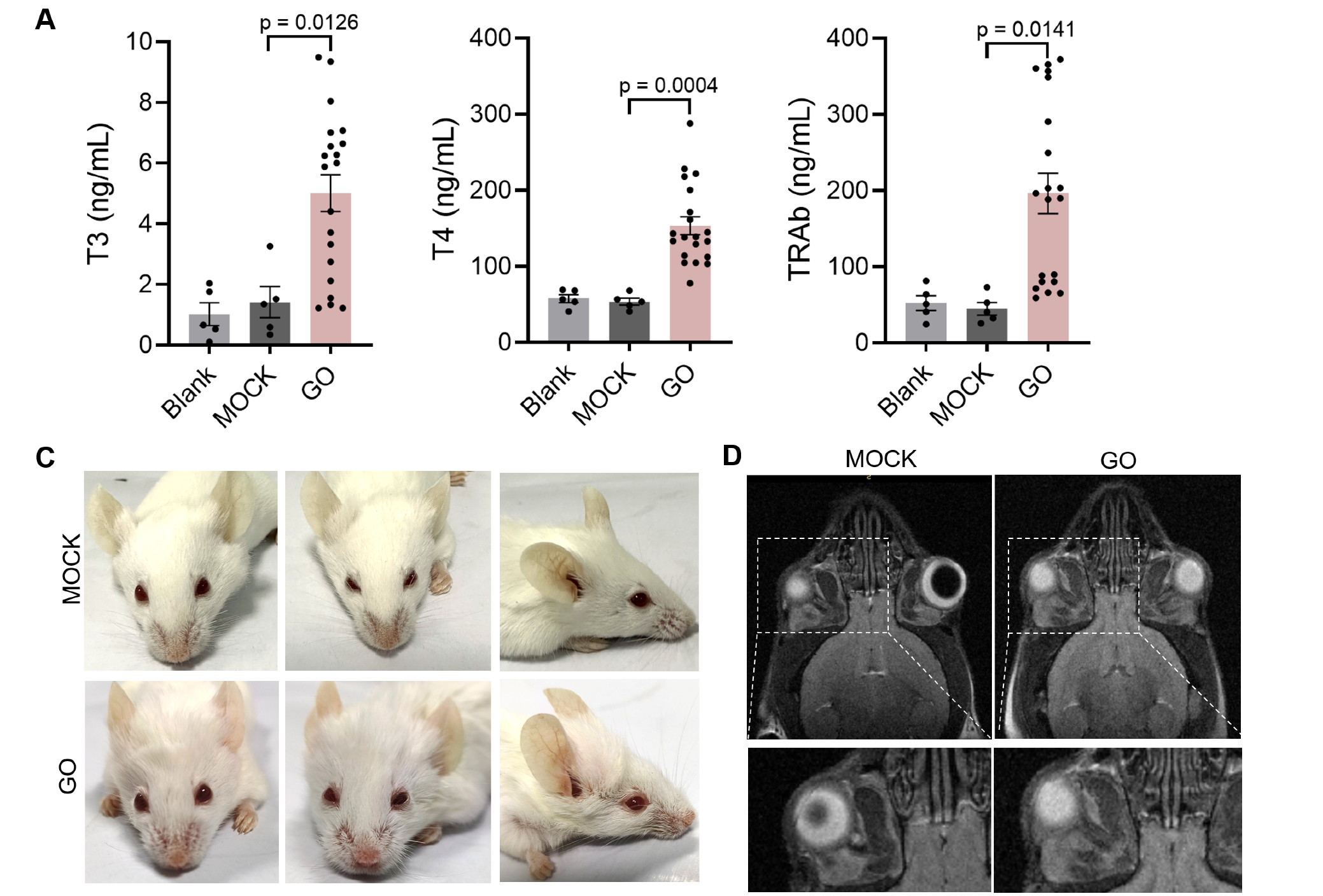


**Figure S11.** Phenotypes of the GO mouse model. A) The concentration of T3, T4, and TRAb in the serum of mice was measured in the Blank group (n = 5), MOCK group (n = 5), and GO group (n = 20) using ELISA. B) Representative images of ocular symptoms in mice from the MOCK group and the GO group. C) Representative T2-weighted Magnetic Resonance Imaging images of mice from the MOCK group and the GO group. The lower images provide an enlarged view of the areas within the white dashed boxes shown in the corresponding upper images. White arrows denote the extraocular muscle. All data are represented as mean ± SEM. Two-tailed unpaired Student’s t-tests (A) were used to calculate P values. Accurate P values are listed in the figures.


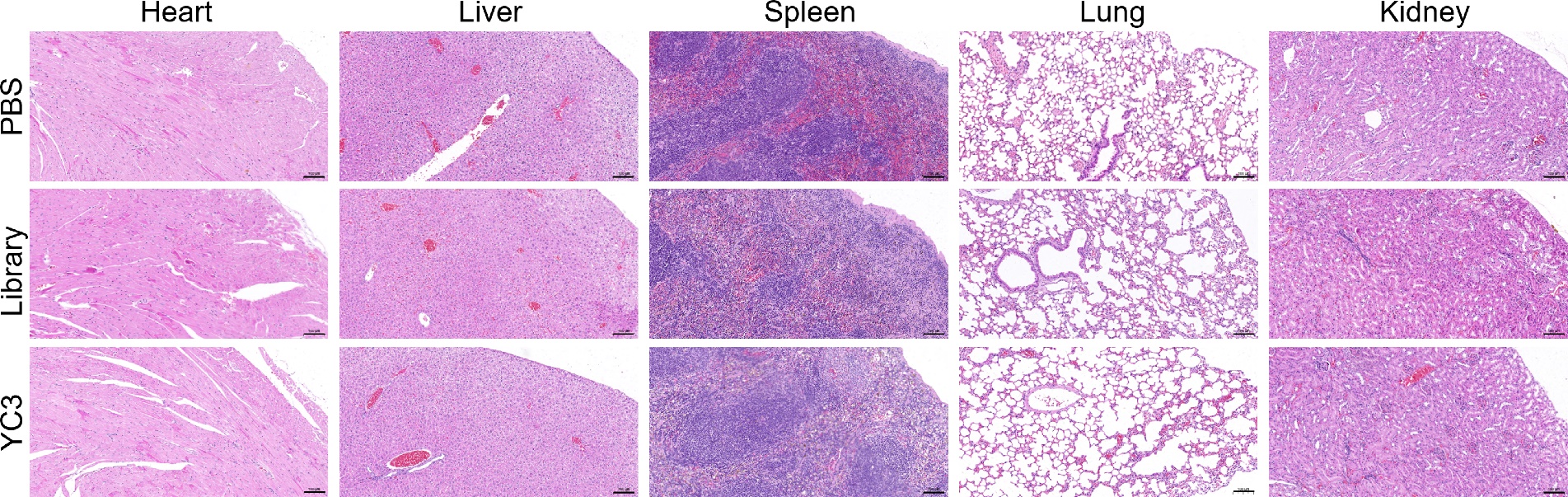


**Figure S12.** Representative H&E staining of heart, liver, spleen, lung, and kidney tissues from GO mice treated with PBS, modified aptamer Library, and modified aptamer YC3 (n = 5). Scale bar: 100 μm.

**References**

1. Zhang YB, Zhang H, Sun X, et al. Nucleic acid aptamer controls mycoplasma infection for inhibiting the malignancy of esophageal squamous cell carcinoma. *Molecular Therapy*. Jun 1 2022;30(6):2224-2241. doi:10.1016/j.ymthe.2022.02.018

2. Li X, Wang T, Xie T, et al. Aptamer-Mediated Enrichment of Rare Circulating Fetal Nucleated Red Blood Cells for Noninvasive Prenatal Diagnosis. *Analytical Chemistry*. 2023;95(12):5419-5427. doi:10.1021/acs.analchem.3c00115

3. Zhang Y, Zhang H, Sun X, et al. Nucleic acid aptamer controls mycoplasma infection for inhibiting the malignancy of esophageal squamous cell carcinoma. *Molecular Therapy : the Journal of the American Society of Gene Therapy*. 2022;30(6):2224-2241. doi:10.1016/j.ymthe.2022.02.018

4. Neumann S, Pope A, Geras-Raaka E, et al. A drug-like antagonist inhibits thyrotropin receptor-mediated stimulation of cAMP production in Graves' orbital fibroblasts. *Thyroid*. Aug 2012;22(8):839-43. doi:10.1089/thy.2011.0520

5. Diao J, Chen X, Mou P, et al. Potential Therapeutic Activity of Berberine in Thyroid-Associated Ophthalmopathy: Inhibitory Effects on Tissue Remodeling in Orbital Fibroblasts. *Invest Ophthalmol Vis Sci*. Sep 1 2022;63(10):6. doi:10.1167/iovs.63.10.6

6. Götz M, Nolden M, Maier-Hein K. MITK Phenotyping: An open-source toolchain for image-based personalized medicine with radiomics. *Radiother Oncol*. 2019;131:108-111. doi:10.1016/j.radonc.2018.11.021
